# Maternal inflammation and oxidative stress during pregnancy in relation to early childhood neurodevelopment

**DOI:** 10.1016/j.bbi.2026.106514

**Published:** 2026-02-23

**Authors:** Seonyoung Park, Deborah J. Watkins, Sung Kyun Park, Bhramar Mukherjee, Wei Hao, Gredia Huerta Montañez, Zaira Y. Rosario Pabón, Carmen M. Vélez Vega, José F. Cordero, Akram Alshawabkeh, John D. Meeker

**Affiliations:** a Department of Environmental Health Sciences, University of Michigan School of Public Health, Ann Arbor, MI 48109, USA; b Department of Epidemiology, University of Michigan School of Public Health, Ann Arbor, MI 48109, USA; c Department of Biostatistics, Yale University School of Public Health, New Haven, CT 06510, USA; d Department of Biostatistics, University of Michigan School of Public Health, Ann Arbor, MI 48109, USA; e Department of Civil and Environmental Engineering, Northeastern University, Boston, MA 02115, USA; f Department of Social Sciences, UPR Medical Sciences Campus, University of Puerto Rico Graduate School of Public Health, San Juan, PR 00921, USA; g Department of Epidemiology and Biostatistics, University of Georgia, Athens, GA 30606, USA

**Keywords:** Inflammation, Oxidative stress, Pregnancy, Neurodevelopment

## Abstract

**Background:**

Pregnancy is a critical period for fetal brain development. Previous animal studies suggest that maternal immune activation can lead to long-term neurodevelopmental issues in offspring. Inflammation and oxidative stress are key upstream pathways leading to immune activation, and both can be triggered by multiple environmental and social stressors. However, a limited number of epidemiological studies have examined the associations of maternal inflammation and oxidative stress during pregnancy with child neurodevelopment.

**Methods:**

We used data from the PROTECT birth cohort in Puerto Rico, including 193 mother–child pairs for inflammation analyses and 247 pairs for oxidative stress analyses, to evaluate associations with early childhood neurodevelopment (ages 1 year to 3 years). Maternal serum concentrations of inflammation biomarkers were measured up to two times during pregnancy (median 18 and 27 weeks gestation), while urinary markers of oxidative stress were measured up to three times (median 18, 23, and 27 weeks). Child neurodevelopment was assessed between ages 1 and 3 years using the Battelle Developmental Inventory-2 Spanish edition (BDI-2), which evaluates adaptive, cognitive, communication, personal-social, and motor domains. Linear mixed-effects models with subject-specific random intercepts were used to account for repeated outcome measurements, while adjusting for potential confounders. Additionally, effect modifications by child sex, age, and gestational timing of biomarker measurements were explored.

**Results:**

Multiple associations were observed between maternal inflammation biomarkers and BDI-2 outcomes. For instance, a doubling in maternal matrix metalloproteinase-1 (MMP1) was associated with a 2.16% lower (95% CI: −3.44, −0.87) adaptive domain score, indicating poorer performance in this domain. We also noted sex-specific findings—generally stronger among males—in both the inflammation and oxidative stress analyses. For example, a doubling of free 8-*iso*-prostaglandin-F2α (IsoP) concentrations was significantly associated with lower adaptive (–2.96%, 95% CI: −5.43, −0.49) and personal-social (–3.21%, 95% CI: −5.35, −1.07) domain scores among male children, but not among female children. Several gestational timing- and child-age–specific associations were also identified, particularly in the oxidative stress analyses. Overall, the negative association between maternal oxidative stress and BDI-2 tended to be stronger at the third gestational visit (~27 weeks of gestation) and at older child ages for most observed associations.

**Conclusion:**

These findings suggest that gestational inflammation and oxidative stress may adversely affect early neurodevelopment, with associations potentially modified by gestational timing, child age, and child sex. Future research should prioritize investigating the mediating roles of inflammation and oxidative stress in the relationship between multiple stressors during pregnancy and child neurodevelopment.

## 1. Introduction

Neurodevelopment in early childhood spans across multiple domains, including adaptive, cognitive, communication, motor, and personal-social skills (Alfonso et al., 2020). Pregnancy is a critical period for fetal brain development, during which the intrauterine environment plays a pivotal role in shaping long-term neurodevelopmental outcomes (Rees & Harding, 2004). Experimental studies have demonstrated that maternal immune activation disrupts neuronal development in the embryonic brain, causing acute proliferation defects, increased neuronal differentiation, and altered cortical lamination (McEwan et al., 2023). Furthermore, animal models have shown that maternal immune activation during pregnancy leads to offspring exhibiting behavioral phenotypes reminiscent of ASD (McEwan et al., 2023; Minakova & Warner, 2018). From animal studies it has also shown that maternal immune activation induces epigenetic changes in the fetal brain (Connor et al., 2012), neuroinflammation (Cieślik et al., 2020), and behavioral problems in offspring (Sal-Sarria et al., 2024), and similar effects may extend to humans. Early identification of risk factors for neurodevelopmental delays in early childhood (i.e., toddlerhood) is crucial, as delays in this period are often predictive of persistent developmental challenges as the child grows (Oldenburg et al., 2020).

Oxidative stress and inflammation may work as key upstream pathways leading to immune activation, and both can be triggered by multiple stressors. Oxidative stress arises when the production of reactive oxygen species (ROS) exceeds the neutralizing capacity of antioxidants, leading to imbalance in antioxidation system (Lushchak, 2014). These excessive ROS can structurally alter proteins and genes, setting off signaling pathways that stimulate inflammation (Lugrin et al., 2014; Wadley et al., 2013), which prompt immune cells to release cytokines and chemokines (Chatterjee, 2016). Epidemiological evidence increasingly links various stressors to the activation of inflammation and oxidative stress pathways. Such stressors include psychosocial challenges (e.g., economic hardship, minority status, or life events such as natural disasters) (Eick et al., 2018; Hänsel et al., 2010; Mocayar Marón et al., 2019) and environmental exposures (e.g., air pollution, chemicals in consumer products, or contaminated foods) (Ghezzi et al., 2018; Münzel & Daiber, 2018). Understanding the relationship of maternal oxidative stress and inflammation with child neurodevelopment will provide insight into the linkage between multiple stressors experienced during pregnancy with child neurodevelopment. However, only a limited number of epidemiological studies have examined the role of maternal inflammation and oxidative stress during pregnancy on childhood neurodevelopmental outcomes, and none have investigated both mechanisms together.

Susceptibility to prenatal inflammatory and oxidative stress exposures can vary markedly by child sex and by the gestational timing of these exposures. Experimental studies have shown that male placentas make fewer compensatory adjustments in gene and protein expression in response to maternal immune activation (Clifton, 2010), potentially leading to more pronounced disruptions in neuronal proliferation and differentiation. Still, both animal and human studies evaluating sex-specific effects of maternal immune activation on brain development have produced mixed results (Rana et al., 2012). Moreover, animal research indicates that embryonic neurogenesis outcomes are significantly influenced by the timing of maternal immune activation induction (McEwan et al., 2023). This suggests that gestational timing of maternal inflammation and oxidative stress may modify the relationship between these maternal pathways and child neurodevelopment, yet few studies have directly investigated this.

Therefore, the objective of this study was to investigate the associations of maternal inflammation and oxidative stress biomarkers during pregnancy with neurodevelopmental outcomes in toddlers aged 1 to 3 years. We also sought to assess susceptibility by child sex and by the gestational timing of maternal inflammation and oxidative stress. Our findings aim to provide insights into the biological mechanisms linking maternal stressors to adverse neurodevelopment in children. Considering that inflammation and oxidative stress are key biological pathways activated by multiple stressors, reducing such exposures is a critical intervention target, particularly in communities facing high socioeconomic and environmental burdens. Mitigating these pathways could optimize early childhood development, carrying significant public health implications.

## 2. Methods

### 2.1. Study participants

This study sample is a subset of the PROTECT prospective birth cohort, which has recruited pregnant women from prenatal clinics and hospitals in northern Puerto Rico since 2010. The PROTECT cohort was initially established to investigate association between environmental chemical exposure and the risk of preterm birth (Cantonwine et al., 2014; Meeker et al., 2013). Due to multiple environmental and social stressors such as chemical contamination, and compromised drinking water, higher poverty rates, and the legacy of colonial history, this population faces elevated risks for gestational inflammation, oxidative stress, and adverse child health outcomes. Study participants were recruited in the first or early second trimester of pregnancy (14 ± 2 weeks’ gestation) and eligible if they 1) were between 18 to 40 years old, 2) lived in the Northern karst region, 3) did not use oral contraceptives 3 months prior to pregnancy, 4) did not use *in vitro* fertilization to become pregnant, and 5) did not have known medical/obstetric complications. Detailed information about the cohort was described in previous studies (Cantonwine et al., 2014). Participants provided urine samples at three time points—visit 1 (median 18 weeks), visit 2 (median 23 weeks), and visit 3 (median 27 weeks)—and blood samples at two time points (visits 1 and 3). In addition, demographic and socioeconomic information was collected through a detailed questionnaire administered at visit 1 (Meeker et al., 2013).

In 2016, the Center for Research on Early Childhood Exposure and Development in Puerto Rico (CRECE) began to track the PROTECT-born children. The CRECE followed children through 4 years of age to monitor developmental status and administered neurodevelopmental assessments at various age points (Manjourides et al., 2020). This study included all the PROTECT mother–child pairs that met both eligibility criteria: (1) at least one prenatal measurement of inflammation or oxidative-stress biomarkers is available, and (2) at least one measurement of child neurodevelopment between ages 1 and 3 years is available. This study was approved by the research and ethics committees of the University of Michigan School of Public Health, University of Puerto Rico, and Northeastern University. All methods reported in this study were performed in accordance with relevant guidelines and regulations imposed by those institutions. All study participants provided full informed consent prior to participation.

### 2.2. Inflammation and MMP biomarker measurements

In our analyses, we included multiple serum biomarkers of inflammation: C-reactive protein (CRP), matrix metalloproteinase-1 (MMP1), −2 (MMP2), −9 (MMP9), intracellular adhesion molecule (ICAM), and vascular cell adhesion molecule (VCAM). These biomarkers were selected to provide a multidimensional snapshot of maternal inflammation during pregnancy, encompassing both systemic and localized signals. Briefly, CRP is a systemic indicator of inflammation (Ballantyne & Nambi, 2005), whereas MMPs are proteins involved in extracellular matrix remodeling and in regulating pro-inflammation cytokines during the inflammation response (Lee & Kim, 2022). ICAM and VCAM mediate inflammation by promoting leukocyte migration to inflammation sites (Singh et al., 2023).

Blood samples were obtained during two visits: visit 1 (median of 18 weeks gestation) and visit 3 (median of 27 weeks gestation). Serum samples were analyzed for inflammation biomarkers using a customized Luminex assay from Invitrogen. The assay followed the manufacturer’s protocol with some modifications, including an overnight incubation at 4 °C with shaking. Prior to the assay, most targets required dilution: CRP was diluted 2000-fold; MMP2 and MMP9 were diluted 50-fold; ICAM and VCAM were diluted 200-fold; MMP1 required no dilution. Raw data were collected using a Luminex-200 plate reader with xPonent software and analyzed using Milliplex analyst (5.1.0.0). Samples were run in duplicate, and the average of duplicate measures was taken. The assays were performed at the Rogel Cancer Center’s Immune Monitoring Shared Resource Center at the University of Michigan. A seven-parameter standard curve, based on measurements of 8 standard concentrations provided by the manufacturer, was used to convert optical density values into concentrations (ng/mL). The highest standard concentration was removed from the standard curve if the percent recovery fell outside the ideal range of 70–130%. More details on the measurement of inflammatory biomarkers in PROTECT are described in our previous study (Lee et al., 2023). Samples measured below the lower limit of detection (LOD) were assigned a value of the LOD divided by the square root of 2 (MMP1, n = 1; MMP2, n = 1), and samples measured above the upper LOD were assigned the upper LOD value (CRP, n = 5), similar to the approach used in previous studies (Kim et al., 2022; Lee et al., 2023).

### 2.3. Oxidative stress biomarker measurements

Urine samples were collected during the three prenatal study visits: visit 1 (median of 18 weeks gestation), visit 2 (median of 23 weeks gestation), and visit 3 (median of 27 weeks gestation). Free 8-*iso*-prostaglandin-F2α (IsoP), and prostaglandin-F2α (PGF2α) were analyzed using stable isotope dilution gas chromatography-negative ion chemical ionization-mass spectrometry (GC/MS) or liquid chromatography-mass spectrometry (LC/MS) at the Eicosanoid Core Laboratory at Vanderbilt University Medical Center (Nashville, TN).

As IsoP can arise from both chemical free radical oxidation and inflammation related enzymatic lipid peroxidation pathways, it is not solely an oxidative stress marker (Van’t Erve et al., 2016). Thus, we differentiated between the absolute concentration of IsoP derived from chemical lipid peroxidation (referred to as absolute chemical lipid peroxidation; aCLP) and that derived from prostaglandin-endoperoxide synthases (referred to as absolute prostaglandin h-synthase; aPGHS), along with their respective fractions (fCLP and fPGHS). The method for quantification was outlined by Van’t Erve and colleagues (Van’t Erve et al., 2016). In summary, the method utilized the ratio of IsoP to PGF2α, which markedly differs between chemical and enzymatic lipid peroxidation pathways. All oxidative stress marker concentrations included in our analyses were measured above the LOD. To adjust for urinary dilution, all concentrations of urinary oxidative stress biomarkers were corrected using specific gravity. This correction was calculated using the formula P_C_ = P [(SG_m_ − 1) / (SG_i_ − 1)], where Pc represents the specific gravity-corrected biomarker concentration (in ng/mL), P is the measured biomarker concentration, SG_m_ is the median specific gravity value of the study population (1.019), and SG_i_ is the specific gravity value for each individual (Meeker et al., 2009).

### 2.4. Neurodevelopmental assessments

Child neurodevelopmental status was assessed using the Battelle Developmental Index-2 Spanish edition (BDI-2) between age of 1 year and 3 years. The BDI-2 is a clinician-administered assessment that evaluates age-specific abilities across five domains—adaptive, cognitive, personal-social, communication, and motor—using scripted interviews and structured play-based activities (Bliss, 2007). Each domain is composed of one to three subdomains depending on age, and the BDI-2 total score represents a composite of all domain scores. All domains and total scores were assessed at ages 1 and 2 years, with the Motor domain score additionally measured at 3 years. For scoring, subdomain raw scores are first converted into scaled scores (mean = 10, SD = 3) using age-specific conversion tables (per 1-month intervals for children under 2 years and per 3-month intervals for children aged 2 years and older). These scaled scores are summed within each domain and then converted into Developmental Quotient (DQ) scores for each domain and for the total score. The DQ scores are norm-based (mean = 100, SD = 15), with lower scores reflecting poorer performance and potential developmental delay. In our analyses, we included DQ scores for the five domains and the total score measured between ages 1 and 2 years, along with the Motor domain DQ score measured at age 3 years.

### 2.5. Statistical analyses

Distributions of maternal inflammation biomarkers and oxidative stress biomarkers were assessed prior to analyses. Since maternal inflammation and oxidative stress biomarker concentrations were right-skewed, natural log-transformation was employed. To assess temporal variability of inflammation and oxidative stress biomarker concentration across gestational visits, we calculated the Intraclass Correlation Coefficient (ICC) of log-transformed biomarker concentration using the ‘psych’ package in R. Additionally, we calculated Spearman correlation coefficients to assess correlations in biomarker concentrations across gestational visits. We also examined the distribution of BDI-2 domain scores by child age group. Additionally, we estimated Spearman correlations among BDI-2 domains within each age group (1 year and 2 years). Correlations were not estimated for the 3-year group because only the Motor domain was measured.

Potential confounders considered were maternal age, maternal education, pre-pregnancy BMI, marital status, exposure to environmental tobacco smoke during pregnancy, alcohol consumption during pregnancy and lifetime, and the number of previous pregnancies. Covariates were selected based on a priori knowledge and effect on main estimates (Curry et al., 2008; Eick et al., 2018; Jiménez-Osorio et al., 2023; Keenan-Devlin et al., 2022). The final models were adjusted for maternal age (continuous), maternal education (categorical; high school, GED, or less; some college; Bachelors or higher), pre-pregnancy BMI (continuous), child age group (categorical), child age in months (continuous) standardized (centered and scaled) within each age group, and child sex. Given the low proportion of missing covariates in each analysis sample (~6%), our analyses were conducted using complete-case data. To assess potential bias from missing covariate data, we repeated the analyses using 20 imputed datasets generated with the mice package in R for multiple imputation (Zhang, 2016). In these analyses, missing outcome data were excluded rather than imputed.

We fitted several models to examine associations between inflammation or oxidative stress biomarker and BDI-2 outcomes across four analytic contexts: (1) the overall sample, (2) child sex, (3) child age group, and (4) by gestational timing of biomarker measurement. To account for repeated outcome measurements in contexts 1, 2, and 4, linear mixed-effects models with participant-specific random intercepts were employed. This approach efficiently handles unbalanced follow-up by utilizing all available data points; while children with repeated measures informed the estimation of within-person correlation, those with a single observation contributed to the estimation of fixed effects via partial pooling (Gelman & Hill, 2007). Critically, the linear mixed effects model framework imposes no constraints regarding the number of observations per individual beyond those of general ordinary least squares (OLS) regression (Wiley & Rapp, 2019). If the total number of observations is sufficient to support fixed-effect estimation in an OLS model, the linear mixed effects model remains viable and provides more robust standard errors by explicitly partitioning between- and within-child variance. In contrast, for age-group–stratified models in the child age group analysis (context 3), standard linear regression models were used as these analyses did not include repeated outcome measurements. In our analytical framework, the overall model and child sex–stratified models were considered primary analyses, whereas analyses stratified by child age group or gestational timing of biomarker measurements were treated as supplementary. Given the exploratory nature of this study, we did not adjust for multiple comparisons.

Details of each model are as follows. (1) Overall model: Linear mixed-effects models were fit in the overall sample with gestational geometric mean of biomarker concentrations as the predictor and repeatedly measured BDI-2 DQ scores as the outcome; gestational geometric means of concentrations was used to reflect overall inflammation and oxidative stress across pregnancy, and subject-specific random intercepts were included to account for within-child correlation. (2) Child sex models: To explore heterogeneity of associations by child sex, linear mixed-effects models were fit with gestational geometric mean of biomarker concentrations as the predictor and repeatedly measured BDI-2 DQ scores as the outcome, including a biomarker × child sex interaction term and subject-specific random intercepts. Only the *p-value* for the interaction term from this model was reported. To facilitate interpretation, we subsequently fit child-sex-stratified linear mixed-effects models without interaction terms, and effect estimates from the stratified models are presented as child sex–specific effects. (3) Child age models: To explore heterogeneity of associations by child age group, linear mixed-effects models were first fit with gestational geometric mean of biomarker concentrations as the predictor and repeatedly measured BDI-2 DQ scores as the outcome, including a biomarker × child age group (categorical; 2 years or 3 years) interaction term and subject-specific random intercepts. Only the *p-value* for the interaction term from this model was reported. Then, age-group–stratified linear regression models without interaction terms were then run, with gestational geometric mean of biomarker concentrations as the predictor and a single (non-repeated) BDI-2 score as the outcome. The effect estimates from the stratified models were presented as age-group-specific effects. (4) Gestational timing models: To evaluate gestational timing–specific associations, linear mixed-effects models were first fit using gestational visit–specific biomarker concentrations as the predictor and repeatedly measured BDI-2 DQ scores as the outcome, including a biomarker × gestational visit (categorical; visit 2 or visit 3) interaction term and subject-specific random intercepts. The interaction models were fit using long-format data, such that each observation contributed only the biomarker concentration measured at the corresponding gestational visit. Therefore, biomarker concentrations from multiple visits were not included simultaneously as covariates. Similar to Models 2 and 3, only the *p-value* from the interaction term was reported. For easier interpretation, gestational visit–stratified linear mixed-effects models without interaction terms were fit using gestational visit–specific biomarker concentrations as the predictor and repeatedly measured BDI-2 DQ scores as the outcome. Then, effect estimates from the visit-stratified models are presented as gestational visit–specific effects.

The regression results were converted to percent change in BDI-2 score compared to the median score per doubling in maternal biomarker concentration using following formula: $\%\;\text{difference}\;in\;y=[(ln2\;*\hat{\beta })/\text{median}\;of\;y]*100$, where ln2 is natural log 2, $\hat{\beta }$ is the estimated coefficient from the regression model, and median of y is the median of each outcome variable across all time points.

## 3. Results

### 3.1. Study population

In this present study, we included mother–child pairs who had data on maternal inflammation biomarkers (N = 193), or maternal oxidative stress biomarkers (N = 247) and child neurodevelopmental outcomes measured at least one age point (1 year, 2 years, or 3 years), with 179 pairs included in both analysis groups. Sample sizes varied slightly across biomarkers and BDI-2 domains due to differences in data availability and completeness of covariates. Among mother–child pairs with inflammatory biomarker data and complete covariate information, 113 children had one adaptive score measurement and 29 had two measurements; for the cognitive domain, 111 had one measurement and 26 had two measurements; and for the communication domain, 113 had one measurement and 30 had two measurements. For the motor domain, 120 children had one measurement, 42 had two measurements, and 10 had three measurements. For the personal–social domain, 112 children had one measurement and 28 had two measurements, while for the total BDI-2 score, 110 had one measurement and 22 had two measurements. Among mother–child pairs with oxidative stress biomarker data and complete covariate information, 156 children had one adaptive score measurement and 31 had two measurements; for the cognitive domain, 148 had one measurement and 28 had two measurements; and for the communication domain, 156 had one measurement and 32 had two measurements. For the motor domain, 161 children had one measurement, 46 had two measurements, and 10 had three measurements. For the personal–social domain, 152 children had one measurement and 30 had two measurements, while for the total BDI-2 score, 143 had one measurement and 24 had two measurements. Additional details on sample sizes by age group are provided in Supplementary Table S4.

A detailed description of the demographics of this study population is summarized in Table 1. Briefly, in both inflammation and oxidative stress analyses groups, the majority of women were under of 30 years of age (58.1% and 61.5%, respectively), had Bachelor’s or higher degree (50.3% and 50.6%, respectively), were currently employed (72.5% and 69.2%, respectively), had a pre-pregnancy BMI under 25 (47.7% and 47%, respectively), were married (56% and 55.9%, respectively), had an annual household income less than 30 k (53.3% and 53%, respectively), had never used or were not consuming alcohol during pregnancy (95.3% and 96.8%, respectively), and were nulliparous (42% and 42.9%, respectively). These demographic characteristics are comparable to those of overall PROTECT cohort and detailed demographic information for the overall PROTECT cohort has been published elsewhere (Ferguson et al., 2019).

### 3.2. Distributions of biomarkers and BDI-2 scores

Distribution of inflammation biomarkers, CRP, MMP1, MMP2, MMP9, ICAM, and VCAM, by each gestational visit are shown in Table 2. All inflammation markers demonstrated moderate (ICCs = 0.5 – 0.75) to good (ICCs = 0.75 – 0.90) reliabilities, indicating greater consistency of measurements within individuals over time. Spearman correlations of inflammatory biomarker concentrations measured across different gestational visits are illustrated in Supplementary Fig. S1a. Overall, inflammatory biomarkers exhibited moderate (ρ = 0.3–0.7) to high (ρ > 0.7) correlations across gestational visits, indicating moderate to strong temporal stability of inflammatory status during pregnancy. The distributions of maternal inflammation biomarkers by demographics and child age and sex are summarized in Supplementary Table S2. In general, MMPs showed similar distribution across maternal characteristics, including age, education, current employment status, marital status, household income, alcohol use, or parity. However, higher ICAM levels were observed in participants who were younger and had lower education (*p-value* = 0.007 or 0.043, respectively). VCAM levels exhibited a non-linear trend with age (*p-value* = 0.03), with elevated values in both the 18–24 and 35–41 age groups, and were also higher among participants with low education (*p-value* = 0.055). In general, maternal inflammation biomarker distributions were comparable across child sex, while VCAM and MMP1 distributions were different by child age groups at follow-up (*p-value* = 0.017 or 0.021, respectively).

Distribution of oxidative stress biomarkers – PGF2a, IsoP, fPGHS, fCLP, aPGHS, and aCLP – by each gestational visit is shown in Table 2. Unlike inflammation biomarkers, the ICCs of most oxidative stress biomarkers are relatively low (< 0.5), indicating greater within individual variability of these markers over time. Spearman correlations of oxidative stress biomarker concentrations measured across different gestational visits are presented in Supplementary Fig. S1b. Overall, oxidative stress biomarkers exhibited low (ρ < 0.3) to moderate (ρ =0.3–0.7) correlations across gestational visits, indicating greater temporal variability and less stability across gestational visits compared with inflammatory biomarkers. The distributions of maternal oxidative stress biomarkers by demographics and child age and sex are summarized in Supplementary Table S3. Briefly, PGF2α concentrations exhibited a non-linear trend across maternal age groups (*p-value* < 0.01), with elevated levels in both the 18–24 and 30–34 age groups, and higher levels observed in participants who had elevated pre-pregnancy BMI (*p-value* = 0.01). IsoP levels were highest in the 18–24 age group, followed by the 30–34 group (*p-value* = 0.01), and were also elevated among participants with lower education (*p-value* < *0.01)*, those not currently employed (*p-value* < *0.01)*, and those with higher pre-pregnancy BMI (*p-value* < *0.01)*.

Distributions of BDI-2 domain scores by age group are summarized in Supplementary Table S4. In general, the mean scores decreased at later visits. We also calculated correlations between different BDI-2 domain scores in inflammation or oxidative stress sample separately (Supplementary Fig. S2). Within each age group, correlations between domains were generally low to moderate, whereas the Total score showed moderate–high correlations with most domains (Supplementary Fig. S2a–b). Correlations were also modestly stronger in the 2-year group than in the 1-year group. Correlations between age groups were moderate for the Communication, Motor, and Total scores in the inflammation analysis sample, and for the Communication and Motor domains in the oxidative stress analysis sample (Supplementary Fig. S2c–d). In contrast, low between–age group correlations were observed for the Adaptive, Cognitive, and Personal–Social domains. These patterns may reflect the relatively small number of repeated measurements and within-child variability over time in the study population.

### 3.3. Maternal inflammation and children’s neurodevelopment

**Fig. 1.**
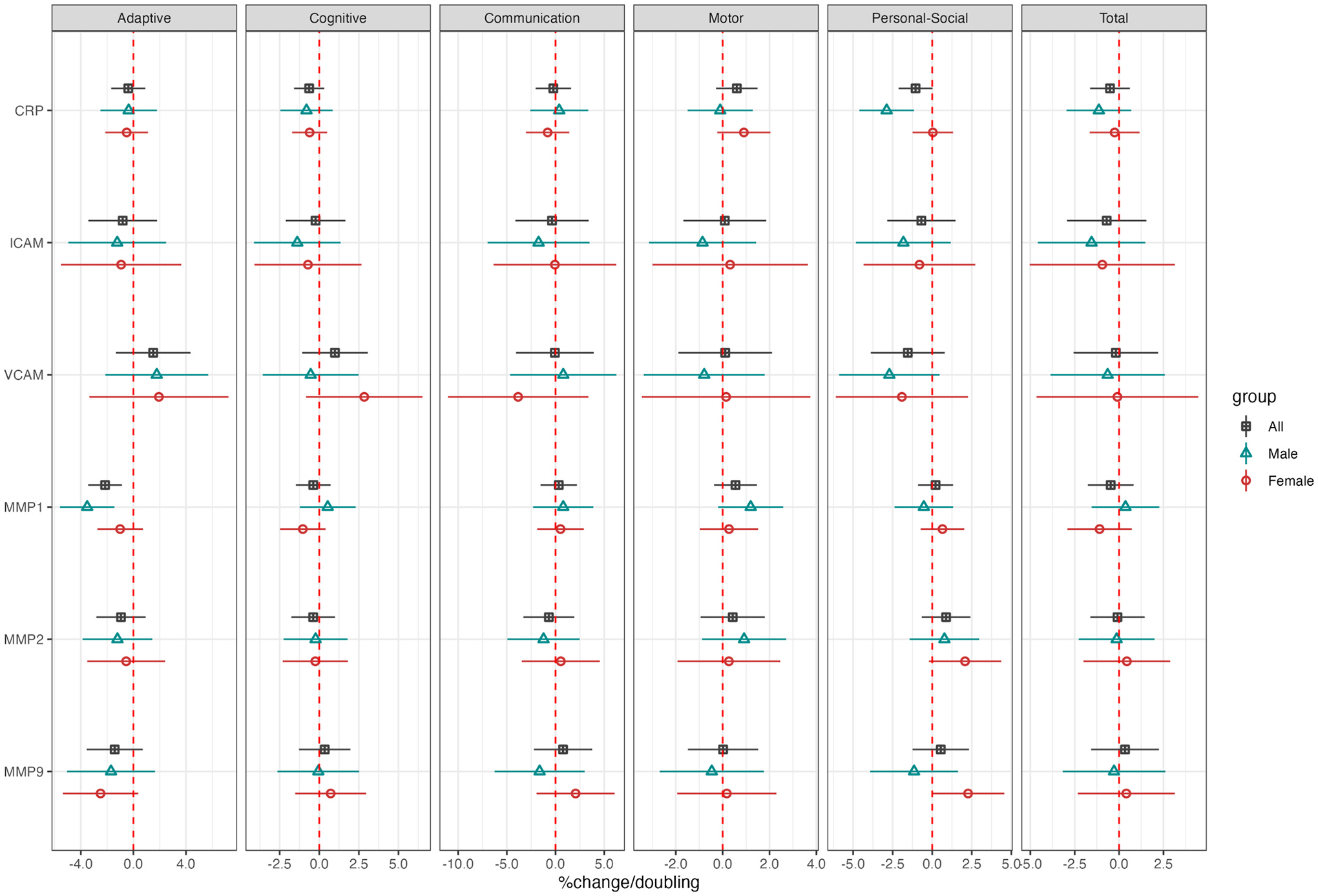
Percent change in BDI-2 DQ scores for a doubling in the gestational geometric mean of maternal inflammation biomarker concentrations by child sex. BDI-2 domain scores were measured at approximately 1 and 2 years of age, with an additional Motor-domain assessment at 3 years. Overall estimates were obtained from linear mixed-effects models with the geometric mean of the maternal biomarker as the predictor and repeated BDI-2 scores as the outcome, adjusted for maternal age, maternal education, pre-pregnancy BMI, child age group, standardized child age in months within each age group, and child sex. Sex-specific estimates were derived from stratified models adjusted for the same covariates, except child sex. The numbers of participants and observations by child sex are following N_all_ = 193 (266), N_girls_ = 93 (134), N _boys_ = 99 (131), N _missing_ = 1 (1).

In overall linear mixed effects models, we observed that a doubling of maternal CRP concentration was marginally associated with a 1.05% lower (95%CI: −2.12, 0.02) than the median of the personal-social domain score, and that a doubling increase in MMP1 was associated with a 2.16% lower adaptive domain score (95%CI: −3.44, −0.87) (Fig. 1, Supplementary Table S5), indicating poorer performance in these domains related to elevated maternal inflammation. Child-sex stratified analyses showed, in general, stronger associations among male children compared to female children. Specifically, the association between CRP and the personal-social domain score was negative only among male children but not among female children (−2.88%/doubling, 95%CI: −4.61, −1.15vs. 0.04%/doubling 95%CI: −1.24, 1.32, p-value of interaction term = 0.003). Similarly, an increase in maternal MMP1 concentration was associated with a lower adaptive domain score among male children (−3.52%/doubling, 95%CI: −5.59, −1.45, p-value of interaction term = 0.106) compared to female children (−1.01%/doubling, 95%CI: −2.74, 0.72). The results were robust to missing covariate data, with estimates from the imputed datasets (data not shown) closely aligning with those from the complete-case analyses.

**Fig. 2.**
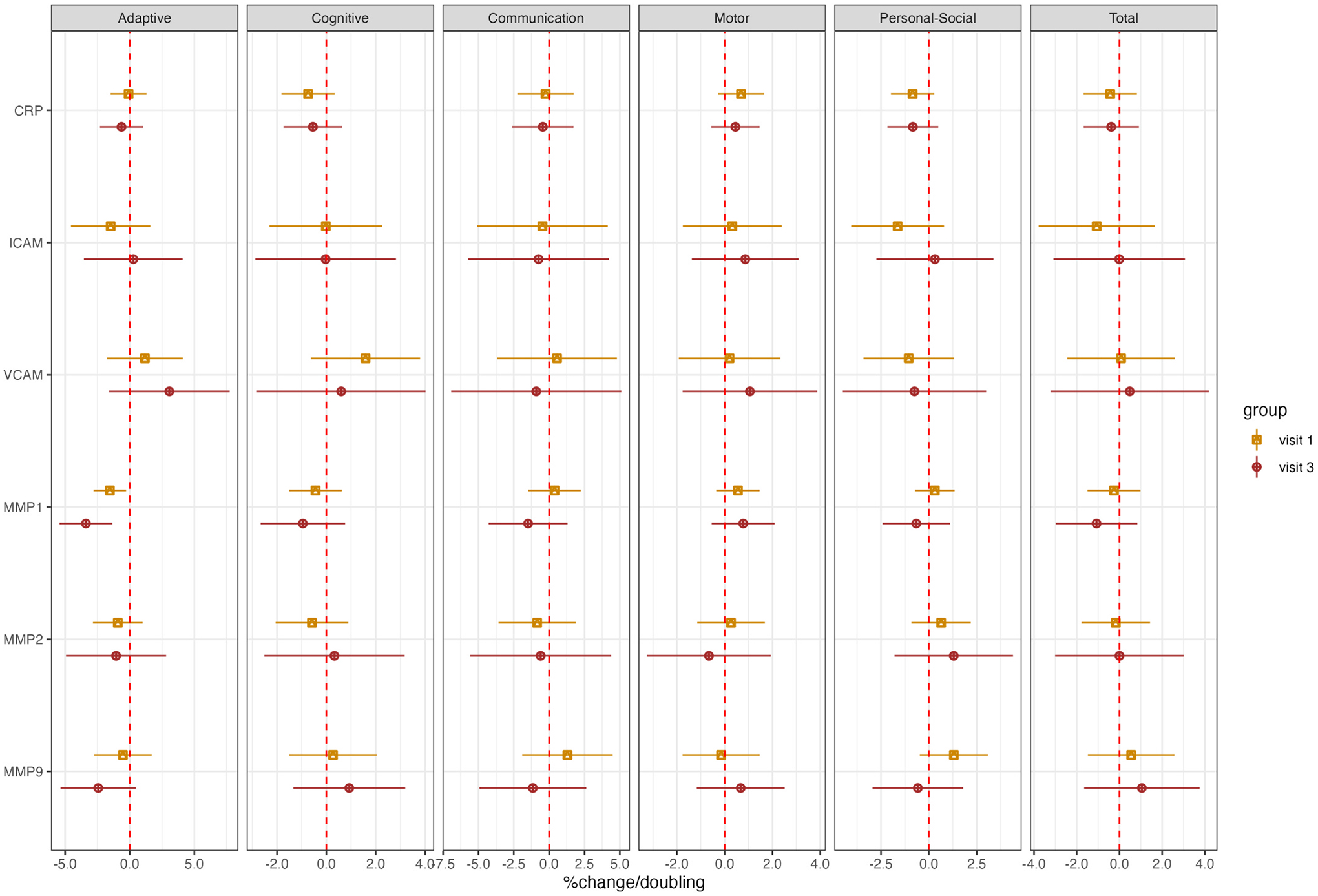
Percent change in BDI-2 DQ scores for a doubling of gestational-visit-specific maternal inflammation biomarker concentrations. BDI-2 domain scores were measured at approximately 1 and 2 years of age, with an additional Motor-domain assessment at 3 years. Overall estimates were obtained from linear mixed-effects models with the gestational-visit-specific maternal biomarker concentration as the predictor and repeated BDI-2 scores as the outcome, adjusted for maternal age, maternal education, pre-pregnancy BMI, child age group, standardized child age in months within each age group, and child sex. Gestational-visit-specific estimates were derived from stratified models adjusted for the same covariates. The numbers of participants and observations in each group are following: N_visit 1_ = 166 (229) and N_visit 3_ = 126 (177).

In gestational-visit-specific analyses adjusted for potential confounders, the observed associations were comparable between gestational visit 1 and visit 3, with a few exceptions. (Fig. 2, Supplementary Table S6). Those exceptions include the stronger association of MMP1 in gestational visit 3 than visit 1 with the adaptive domain score. Specifically, a doubling in MMP1 measured at gestational visit 3 was associated with 3.39% lower adaptive domain score (95%CI: −5.44, −1.35), while associated with 1.54% lower score (95%CI: −2.8, −0.28) at gestational visit 1. Similarly, MMP9 at gestational visit 3 was marginally associated with lower adaptive domain score (−2.44%/doubling, 95% CI: −5.35, 0.48), while at gestational visit 1 the association was null. Still, the general trend of associations was similar across different gestational visits. In addition, we observed several effect modification by child age group (categorical; 2 years or 3 years), based on the interaction term between the two variables – maternal inflammation biomarker concentrations and child age group (categorical; 2 years or 3 years) – as shown in the Supplementary Table S7 and the child-age-stratified results (Supplementary Fig. S3). The association between adaptive domain and MMP1 observed in overall group was primarily driven by assessments at 1 year of age (−3.04%/doubling, 95%CI: −4.66, −1.42, p-value of interaction term = 0.035), while the association of the personal-social domain with CRP was stronger among participants assessed at 2 years of age (−2.24%/doubling, 95%CI: −4.1, 0.37, p-value of interaction term = 0.192). More detailed results are summarized in Supplementary Table S7.

### 3.4. Maternal oxidative stress and children’s neurodevelopment

**Fig. 3.**
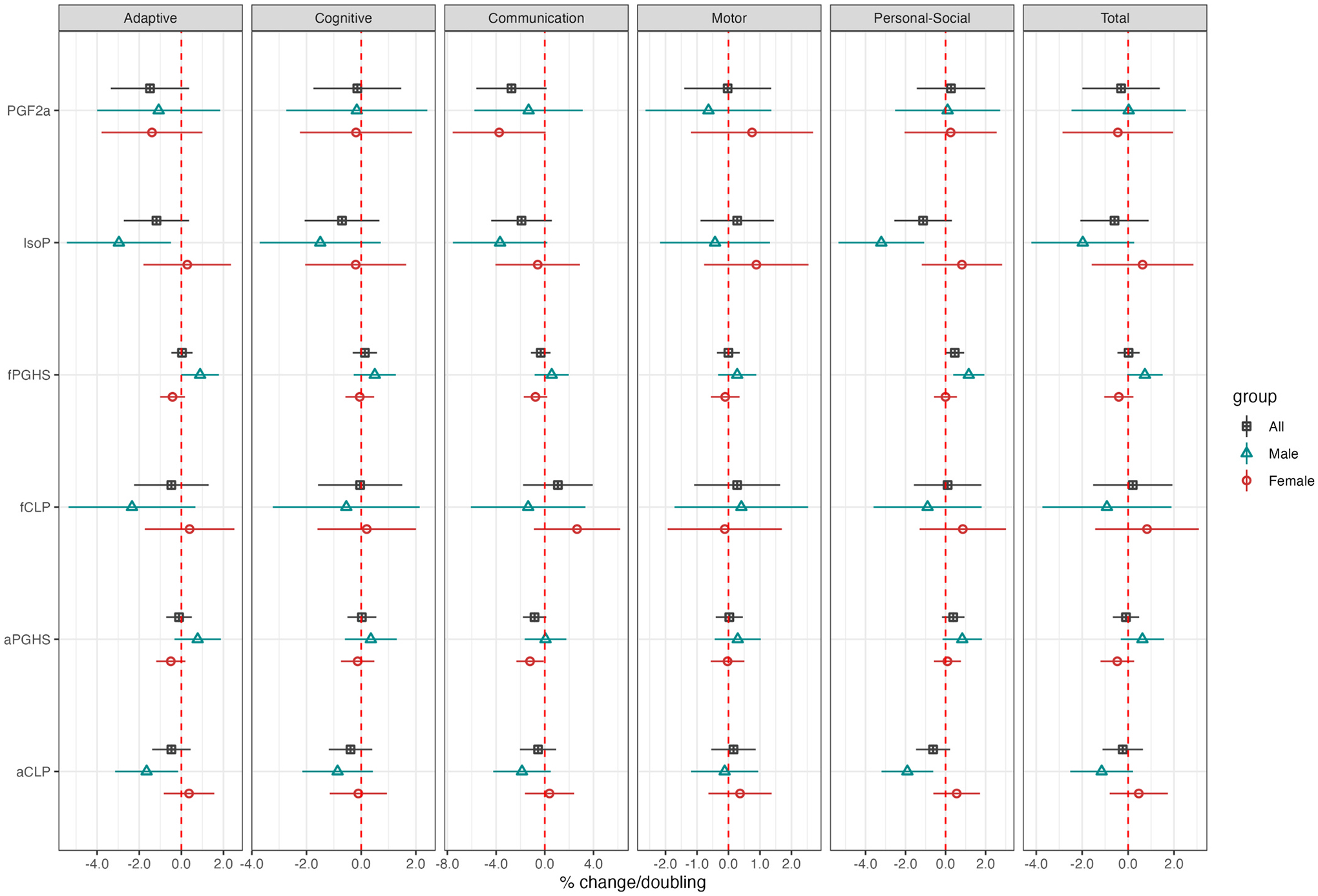
Percent change in BDI-2 DQ scores for a doubling in the gestational geometric mean of specific gravity adjusted maternal oxidative stress biomarker concentrations by child sex. BDI-2 domain scores were measured at approximately 1 and 2 years of age, with an additional Motor-domain assessment at 3 years. Overall estimates were obtained from linear mixed-effects models with the geometric mean of the maternal biomarker as the predictor and repeated BDI-2 scores as the outcome, adjusted for maternal age, maternal education, pre-pregnancy BMI, child age group, standardized child age in months within each age group, and child sex. Sex-specific estimates were derived from stratified models adjusted for the same covariates, except child sex. The numbers of participants and observations by child sex are following N = 247 (321), N _girls_ = 124 (169), N _boys_ = 121 (150), N_missing_ = 2 (2).

In the overall linear mixed effects models using gestational geometric mean of oxidative stress marker levels, we noted only a few marginal associations—for example, a lower communication score was linked to PGF2α (−2.74%/doubling; 95% CI: −5.63, 0.15) (Fig. 3, Supplementary Table S8). We also observed modest inverse relationships between IsoP and most domains, except for the motor domain score. In the sex-stratified analyses, multiple significant associations between oxidative stress biomarkers and BDI-2 scores became more pronounced (Fig. 3). For example, increased IsoP concentration was significantly associated with lower adaptive (−2.96%/doubling, 95%CI: −5.43, −0.49) and personal-social domain scores (−3.21%/doubling, 95%CI: −5.35, 1.07) among male children but not among female children. Similarly, a doubling increase in aCLP was associated with 1.65% decreased adaptive (95%CI: −3.15, −0.15) and 1.91% decrease in personal-social (95%CI: −3.2, −0.62) domain scores only among males. In contrast, we observed that aPGHS was associated with a reduced communication domain score but only among female children (−1.22%/doubling, 95% CI: −2.34, −0.09).

**Fig. 4.**
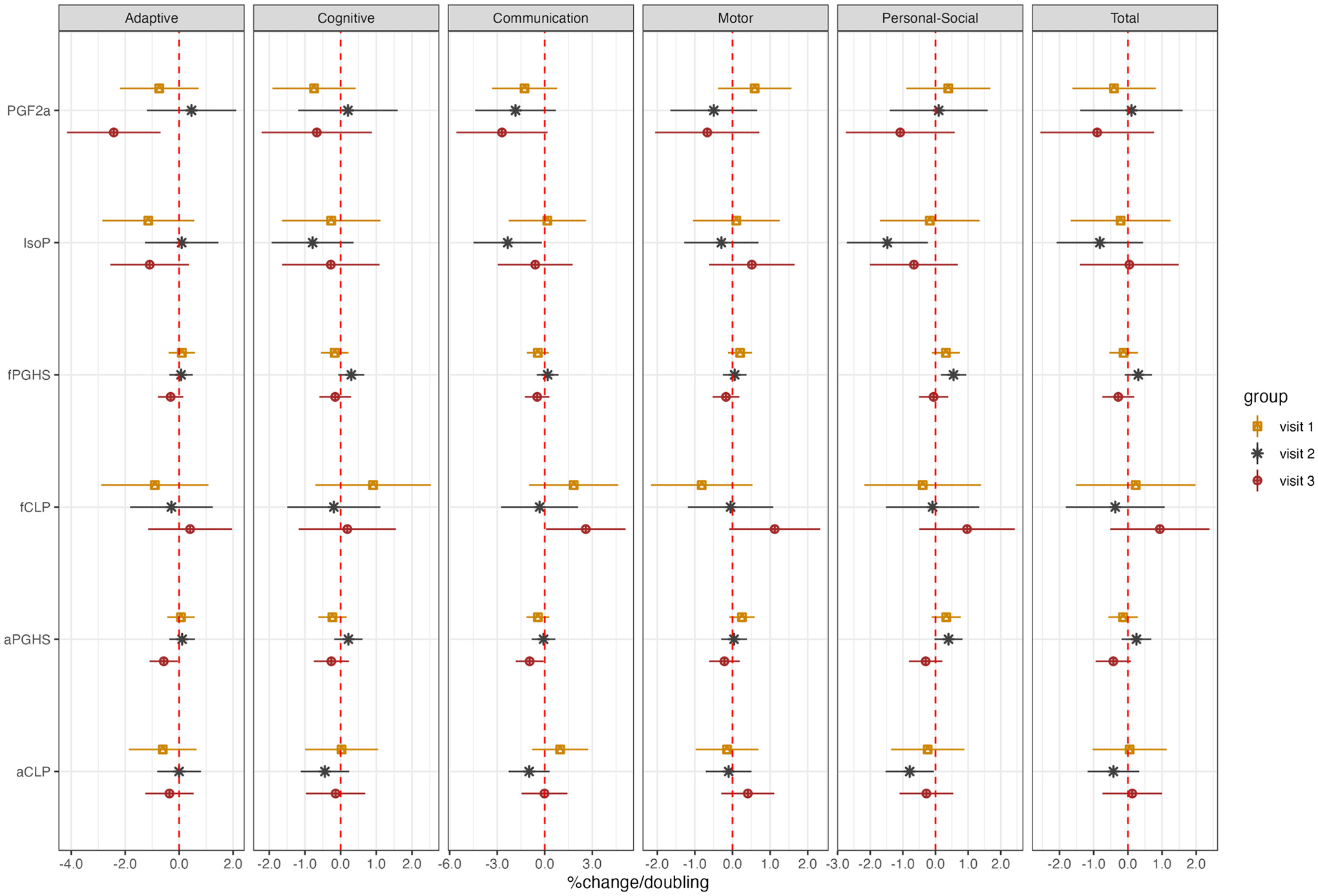
Percent change in BDI-2 DQ scores for a doubling in the gestational-visit-specific maternal oxidative stress biomarker concentrations. BDI-2 domain scores were measured at approximately 1 and 2 years of age, with an additional Motor-domain assessment at 3 years. Overall estimates were obtained from linear mixed-effects models with the gestational-visit-specific maternal biomarker concentration as the predictor and repeated BDI-2 scores as the outcome, adjusted for maternal age, maternal education, pre-pregnancy BMI, child age group, standardized child age in months within each age group, and child sex. Gestational-visit-specific estimates were derived from stratified models adjusted for the same covariates. The numbers of participants and observations by each study visit are following: N_visit 1_ = 195 (255), N_visit 2_ = 203 (265), and N_visit 3_ = 179 (228).

Based on preliminary models including an interaction term, we found no statistically significant interaction between oxidative-stress measurements and gestational visit (Supplementary Table S9). However, gestational-visit–stratified analyses revealed several visit-specific associations after adjustment for potential confounders. (Fig. 4). For example, a doubling in PGF2α concentration and a doubled aPGHS at gestational visit 3 was associated with a 2.42% (95% CI: −4.16, −0.69, p-value of interaction term = 0.791) and 0.57% (95% CI: −1.1, −0.05, p-value of interaction term = 0.785) lower adaptive domain score, respectively, with no significant associations at earlier gestational visits. Similarly, higher aPGHS levels were associated with a lower communication domain score only at gestational visit 3 (−0.95% per doubling, 95% CI: −1.83, −0.07, p-value of interaction term = 0.972). In contrast, the significant negative associations between IsoP concentrations and a lower communication domain score (−2.34% per doubling, 95% CI: −4.49, −0.18, p-value of interaction term = 0.801) and lower personal-social domain score (−1.48% per doubling, 95% CI: −2.72, −0.24, p-value of interaction term = 0.771) were observed only at gestational visit 2. Moreover, we observed several effect modification by child age groups, based on the interaction term between the two variables – maternal oxidative tress biomarker concentrations and child age group (categorical; 2 years or 3 years) – and the child-age-group stratified results (Supplementary Fig. S4, Supplementary Table S10). In general, we observed stronger negative trends in older ages (2 years for other domains, and 3 years for the motor domain) than in the 1 year old group. For example, a doubling in IsoP was associated with lower scores of adaptive (−2.89%/doubling, 95%CI: −5.35, −0.43, p-value of interaction term = 0.107), communication (−5.48%/doubling, 95%CI: −9.75, −1.21, p-value of interaction term = 0.012), and personal-social (−2.62%/doubling, 95%CI: −4.96, −0.29, p-value of interaction term = 0.208) domains measurements at the 2-year visit, but not at the 1-year visit. More detailed results are summarized in Supplementary Table S10.

## 4. Discussion

In this study, we explored the associations between maternal inflammation and oxidative stress biomarkers during pregnancy and child neurodevelopmental outcomes at ages 1 through 3 years. We found that elevated concentrations of several maternal inflammation and oxidative stress markers were associated with lower BDI-2 scores across various domains, suggesting a higher risk of neurodevelopmental delay in multiple areas. We also observed notable child sex-specific and gestational-timing-specific associations. For example, for both inflammation and oxidative stress biomarkers, child sex-specific analyses indicated that several associations were stronger in male children compared to females. In the gestational-visit-stratified analyses, the associations between inflammation biomarkers and BDI-2 scores were generally consistent across gestational visits, whereas the associations with oxidative stress biomarkers were driven primarily by concentrations measured at gestational visits 2 (median 23 weeks gestation) and 3 (median 27 weeks gestation), rather than gestational visit 1 (median 18 weeks gestation). Additionally, our sensitivity analyses revealed a pronounced effect modification by child age in the oxidative stress analyses. Overall, our results provide insights into how maternal inflammation and oxidative stress during pregnancy may contribute to neurodevelopmental challenges in early childhood, and multiple modifying factors of these associations.

Although few epidemiological studies exist, prior research has shown that maternal inflammation and oxidative stress are associated with neurodevelopmental problems in children across a range of ages. For instance, Nazzari et al. (2020) reported that gestational CRP levels were associated with lower cognitive development scores in infants at 12 weeks old. In addition, Lee et al. (2024) found that elevated concentrations of inflammation biomarkers, including CRP and multiple interleukins, in umbilical cord blood were linked to higher odds of delays in motor and communication skills at 24 months. Similarly, Kelly et al. (2022) showed that interleukin-8 (IL-8) concentrations during early (10–18 weeks) and late (32–38 weeks) pregnancy were associated with worse fine-motor and problem-solving skills measured at age two. Furthermore, maternal CRP concentrations during pregnancy, particularly in the third trimester, have been linked to poorer executive function in children aged 4 to 6 years (Morgan et al. (2020)). In a recent study, Eick et al. (2024) found that increased levels of PGF2α were associated with reduced novelty preference, indicating poorer visual recognition memory in children at 7.5 months. Carey et al. (2024) reported that higher levels of IsoP were suggestive of lower cognitive ability scores among 3-year-old children. Moreover, Rommel et al. (2020) used the same oxidative stress marker, IsoP, and reported its association with higher scores on the social responsiveness scale at age 4 years in the TIDES study, suggesting potential issues in social awareness, cognition, and interaction.

Our findings align with previous studies demonstrating associations between maternal inflammation or oxidative stress markers and increased risk of neurodevelopmental delays across multiple domains, while also offering additional insights for several reasons. Our study provides further evidence on the potential role of maternal inflammation and oxidative stress during pregnancy in child neurodevelopment by simultaneously examining biomarkers from both pathways. This approach is particularly valuable given our confirmation of findings in marginalized communities, where pregnant people are more likely to be exposed to multiple stressors. Moreover, we employed multiple derivatives of the oxidative stress marker (IsoP) to distinguish between oxidative stress resulting from chemical lipid peroxidation and that stemming from inflammation-related enzymatic processes. This further enhances our understanding of the biological mechanisms underlying the observed associations.

In our data, PGF2α, IsoP, and aPGHS all exhibited a consistent negative trend in relation to BDI-2 scores. Because PGF2α is primarily generated via enzymatic inflammatory pathways, and aPGHS reflects enzymatically derived IsoP, these associations imply that maternal oxidative stress influences child BDI-2 outcomes through inflammation-related mechanisms. These findings underscore the interconnected roles of maternal inflammation and oxidative stress in shaping child neurodevelopment. Moreover, maternal oxidative stress during pregnancy may directly impact neuronal development (Barron et al., 2021). For instance, experimental studies have demonstrated that placental oxidative stress can hinder the dendritic growth of cortical neurons and the expression of GluN1, which plays a crucial role in intracellular signaling (Scott et al., 2018) (McNearney & Westlund, 2023).

Activated maternal inflammation during pregnancy may impact infant neurodevelopment through several molecular mechanisms. First, proinflammation cytokines such as IL-6 and IL-1β can cross the placenta and enter the fetal bloodstream (Dahlgren et al., 2006; Girard et al., 2010; Zaretsky et al., 2004). Additionally, inflammation in the placenta is associated with a reduction in dendritic processes in the offspring’s brain, which can lead to deficits in learning and memory (Burd et al., 2010; Elovitz et al., 2011). A recent study by Goeden et al. (Goeden et al., 2016) suggested that maternal inflammation during pregnancy may disrupt fetal brain development by increasing serotonin expression in the placenta in mouse models. In addition, inflammation induced immune activation may adversely affect fetal brain development. During pregnancy, maternal tryptophan (TRP) is converted into serotonin (5-HT), which is essential for cell proliferation, migration, and circuit wiring (Bonnin & Levitt, 2011), providing an exogenous source of 5-HT for the fetus. However, maternal immune challenges involving inflammation can upregulate the pathway converting TRP to 5-HT, leading to an accumulation of exogenous 5-HT that negatively affects the endogenous 5-HT necessary for fetal axonal outgrowth (Goeden et al., 2016).

Our observation of stronger associations among males is supported by experimental evidence. Research suggests that the male fetal placenta is more vulnerable to maternal immune activation and inflammation responses than the female placenta (Hunter et al., 2021), which might be due to fewer placental adjustments in gene and protein expression for male fetuses compared to female fetuses (Clifton, 2010). In a recent animal study, Osman et al (2024) examined the impact of maternal immune activation on placental and fetal brain development and discovered that cytokine responses were more prominent in male offspring placenta and brain tissue than in female offspring. Furthermore, male and female placentas exhibit distinct structural and molecular differences, which may explain sex-specific patterns of neurodevelopmental issues related to oxidative stress (Ramiro-Cortijo et al., 2017). For instance, male placentas displayed a stronger prooxidant state, with elevated levels of protein carbonyls, lipid hydroperoxides, and nitrotyrosine, but lower glutathione peroxidase activity, thus more susceptible to maternal oxidative stress compared to female placentas (Stark et al., 2011). Moreover, sex hormones may play a role in child-sex-specific effect of maternal oxidative stress. Sex hormones like estradiol and estriol may function as antioxidants alongside their hormonal roles (Lagranha et al., 2018; Prokai et al., 2005). Since female fetuses have higher circulating levels of these hormones, they may receive greater protection against oxidative stress compared to male fetuses. (Reyes et al., 2006).

In our analysis of inflammation biomarkers, while overall similar trends were observed across gestational visits, some associations were stronger at gestational visit 3 compared to visit 1. We consistently found that oxidative stress biomarkers from inflammation pathways, such as PGF2α and aPGHS, measured at gestational visit 3, showed stronger associations with child neurodevelopment compared to oxidative stress measurements taken at gestational visit 1. Biomarker measurements at gestational visit 1 (~18 weeks) occur in the early second trimester, while gestational visit 3 (~27 weeks) marks the transition between the second and third trimesters. This pattern may be explained by the fact that the period between 24 and 28 weeks of gestation (late second to early third trimester) is critical for fetal brain development. During this time, the organization of the neural network begins, characterized by the outgrowth of nerve fibers, synaptogenesis, and the proliferation and differentiation of neuroglia (Linderkamp et al., 2009). Additionally, the second trimester is generally considered an anti-inflammation phase to support fetal growth, which shifts to a pro-inflammation state in the third trimester in preparation for childbirth (Mor, 2008). As maternal anti-inflammation responses wane in later gestation, the fetus may become more susceptible to inflammation, which could impact brain development. In contrast, other oxidative stress markers, such as IsoP and its non-enzymatic formulation (aCLP, from non-inflammation pathways), showed stronger associations at gestational visit 2 (~23 weeks gestation). These trends may reflect the temporal dynamics of maternal antioxidant and oxidative stress levels during pregnancy. Reactive oxygen species (ROS) production increases over time as the developing fetus demands more oxygen and utilizes fatty acids, with a notable surge during the second trimester (Hussain et al., 2021). Maternal antioxidant levels, which are suppressed during the first trimester to prevent pregnancy failure, increase in the second and third trimesters (Hussain et al., 2021). The stronger associations between oxidative stress measured in the mid-second trimester and neurodevelopmental outcomes may be due to an imbalance between excessive ROS production and insufficient maternal antioxidant capacity during this period.

Though limited studies have examined age-specific findings, some evidence supports our results. For example, Garay et al. (2013) demonstrated that maternal immune activation during pregnancy alters cytokine levels in offspring’s brains in both region- and age-specific manners from a mouse model. Specifically, in the frontal and cingulate cortices, multiple pro- and anti-inflammation cytokines were elevated in early postnatal maternal-immune-activated offspring (7 to 14 postnatal days), corresponding to 9 to 18 months in human age (Dutta & Sengupta, 2016). In contrast, lower cytokine concentrations were observed at later time points (40 postnatal days), roughly equivalent to 40 months in human age (Dutta & Sengupta, 2016). However, there is still limited information on how these associations vary by child age, highlighting the need for further investigation into the underlying molecular mechanisms. Additionally, we observed stronger associations at later ages (2 to 3 years) compared to 1 year, which may be due to certain neurodevelopmental problems becoming more noticeable as children grow older. For instance, the brain continues to grow and reorganize between the ages of 2 and 6, reaching 90% of its adult size by this period (Lenroot & Giedd, 2006), which reflects the variability of neurodevelopment as children age. We found stronger trends of association with delays in adaptive and personal-social skills at 2 years compared to 1 year. However, a more comprehensive evaluation of these skills may be possible in older children, such as during the preschool years (~3 to 5 years) (Bondi et al., 2024). Future research should explore the long-term impact of maternal inflammation and oxidative stress on delays across multiple neurodevelopmental domains in older children to validate and extend these findings.

Our study has several strengths. First, we employed a comprehensive set of biomarkers to assess maternal inflammation and oxidative stress during pregnancy, enhancing our understanding of their potential role in neurodevelopmental delays during early childhood. Additionally, we used the BDI-2, a validated tool for assessing neurodevelopment across multiple domains during the toddler years, which is also suitable for administration in Spanish-speaking populations. We conducted several sensitivity analyses to examine child sex-related vulnerability, identify potential windows of susceptibility using gestational-timing-specific biomarker concentrations, and investigate age-specific associations between child development and maternal biomarkers. Our findings suggest possible biological pathways between gestational exposure to multiple stressors and adverse child developmental outcomes. However, our study has some limitations. First, the sample consisted of pregnant women and their children from marginalized communities in Puerto Rico, where environmental contamination and socioeconomic challenges are significant. While it is an important population to study and offers a setting with various strengths, it may limit the generalizability of our findings. Second, while the third trimester is a critical period for fetal development, we were only able to collect maternal biological samples during the second trimester. Future studies that measure maternal inflammation and oxidative stress across all stages of gestation will provide a more comprehensive understanding. Additionally, measurements of oxidative stress in urine and inflammatory biomarkers in serum prevented direct cross-matrix comparisons in this study. Thus, future studies with parallel measurements across both matrices would help clarify potential matrix-specific effects and strengthen interpretation of associations. Third, our study focused on children aged 1 to 3 years, but neurodevelopment is a continuous process with more advanced cognitive and social skills developing as children grow. Longer follow-up periods will be necessary to assess the long-term associations of maternal inflammation and oxidative stress during pregnancy with persistent neurodevelopmental challenges. Finally, although we conducted a large number of models for exploratory purposes, we did not adjust for multiple comparisons. In addition, our relatively limited sample size restricted statistical power and our ability to formally control for multiple testing. Consequently, future studies with larger sample sizes and appropriate adjustment for multiple comparisons are needed to confirm our findings.

## 5. Conclusion

Our study found that maternal inflammation and oxidative stress biomarkers during pregnancy were associated with higher risk of neurodevelopmental delays across multiple domains in toddlers (ages 1 year to 3 years). Notably, these associations varied by gestational timing, child sex and age, highlighting the nuanced and dynamic nature of these biological processes. The consistent trends observed for both inflammation and oxidative stress in relation to neurodevelopmental outcomes underscore the complex interplay between these maternal factors and child brain development during pregnancy. Future research should prioritize investigating the mediating role of inflammation and oxidative stress in the relationship between prenatal exposure to stressors and child neurodevelopment. Additionally, longitudinal analyses tracking neurodevelopmental trajectories over time, as more data becomes available, will offer deeper insights into how these early biological disruptions may be related to child development throughout early childhood and beyond. Such research could further inform strategies for mitigating environmental risks to optimize early childhood development and represent goals for our future work with the PROTECT cohort.

## Supplementary Material

1

## Figures and Tables

**Table 1:** Demographic characteristics of study population.

		Inflammation Analyses Group N = 193	Oxidative Stress Analyses Group N = 247
Maternal Age (years)	18–24	47 (24.4%)	67 (27.1%)
	25–29	65 (33.7%)	85 (34.4%)
	30–34	50 (25.9%)	58 (23.5%)
	35–41	31 (16.1%)	37 (15%)
Maternal Education	GED or less	24 (12.4%)	31 (12.6%)
	Some college	69 (35.8%)	88 (35.6%)
	Bachelors or higher	97 (50.3%)	125 (50.6%)
	Missing	3 (1.6%)	3 (1.2%)
Currently Employed	No	52 (26.9%)	75 (30.4%)
	Yes	140 (72.5%)	171 (69.2%)
	Missing	1 (0.5%)	1 (0.4%)
Pre-pregnancy BMI	≤25	92 (47.7%)	116 (47%)
	>25 – <30	54 (28%)	64 (25.9%)
	≥30	35 (18.1%)	49 (19.8%)
	Missing	12 (6.2%)	18 (7.3%)
Marital Status	Single	24 (12.4%)	32 (13%)
	Married	108 (56%)	138 (55.9%)
	Cohabitating	59 (30.6%)	74 (30%)
	Missing	2 (1%)	3 (1.2%)
Annual Household Income	<10 k	41 (21.2%)	51 (20.6%)
	10 k – <30 k	62 (32.1%)	80 (32.4%)
	30 k – <50 k	52 (26.9%)	58 (23.5%)
	≥50 k	23 (11.9%)	30 (12.1%)
	Missing	15 (7.8%)	28 (11.3%)
Alcohol Use	Never	89 (46.1%)	118 (47.8%)
	Yes, before pregnancy	95 (49.2%)	121 (49%)
	Yes, currently	8 (4.1%)	7 (2.8%)
	Missing	1 (0.5%)	1 (0.4%)
Pregnancy Number	0	81 (42%)	106 (42.9%)
	1	66 (34.2%)	79 (32%)
	2—5	46 (23.8%)	62 (25.1%)
Child Sex	Female	93 (48.2%)	124 (50.2%)
	Male	99 (51.3%)	121 (49%)
	Missing	1 (0.5%)	2 (0.8%)

**Table 2:** Distribution of maternal inflammation biomarker concentration (ng/mL) and specific gravity adjusted oxidative stress biomarker concentration (ng/mL) among study participants. Values are presented as geometric mean (interquartile range) for gestational geometric mean concentrations and visit-specific concentrations at each gestational visit (Visits 1–3). Gestational age at sample collections were around 18 weeks (visit 1), 23 weeks (visit 2), and 27 weeks (visit 3).

Inflammation Marker	Gestational Geometric Mean	Gestational Visit 1	Gestational Visit 3	ICC	ICC 95% CI
CRP	2721.2 (1331.87, 5833.02)	2839.40 (1452.56, 5357.49)	2669.86 (1175.99, 6366.40)	0.61	(0.52 0.69)
ICAM	641.52 (501.87, 746.17)	625.73 (508.75, 721.12)	672,32 (487.61, 791.34)	0.83	(0.78, 0.87)
VCAM	229.14 (190.91, 302.54)	227.77 (190.34, 294.86)	241.68 (202.91, 312.43)	0.6	(0.5, 0.68)
MMP1	0.29 (0.16, 0.54)	0.275 (0.152, 0.507)	0.334 (0.191, 0.574)	0.75	(0.67, 0.81)
MMP2	10.36 (7.78, 10.71)	10.51 (7.80, 11.04)	9.19 (7.22, 10.55)	0.78	(0.72, 0.83)
MMP9	30.81 (22.55, 46.56)	31.82 (23.26, 47.88)	29.49 (22.39, 43.78)	0.52	(0.4, 0.61)

Oxidative Stress Marker	Gestational Geometric Mean	Gestational Visit 1	Gestational Visit 2	Gestational Visit 3	ICC	95% CI
PGF2a	3.13 (2.19, 4.3)	2.87 (1.82, 4.51)	3.03 (2.16, 4.58)	3.34 (2.3, 5.1)	0.27	(0.19, 0.35)
IsoP	1.56 (1.14, 2.28)	1.62 (1.11, 2.33)	1.52 (1.07, 2.26)	1.52 (1.01, 2.4)	0.32	(0.24, 0.4)
fPGHS	0.12 (0.04, 0.48)	0.1 (0.07, 0.46)	0.12 (0.08, 0.52)	0.15 (0.12, 0.68)	0.31	(0.23, 0.39)
fCLP	0.67 (0.5, 0.91)	0.71 (0.55, 0.94)	0.69 (0.52, 0.95)	0.66 (0.44, 0.96)	0.41	(0.33, 0.49)
aPGHS	0.17 (0.07, 0.52)	0.15 (0.12, 0.62)	0.16 (0.13, 0.6)	0.2 (0.21, 0.65)	0.22	(0.14, 0.31)
aCLP	0.95 (0.67, 1.75)	1.08 (0.73, 1.95)	0.93 (0.63, 1.88)	0.88 (0.54, 1.81)	0.42	(0.34, 0.49)

## Data Availability

The authors do not have permission to share data.

## Appendix A. Supplementary data

Supplementary data to this article can be found online at https://doi.org/10.1016/j.bbi.2026.106514.

## References

[R1] Alfonso VC, Bracken BA, Nagle RJ, 2020. Psychoeducational assessment of preschool children. Routledge.

[R2] Ballantyne CM, Nambi V, 2005. Markers of inflammation and their clinical significance. Atheroscler. Suppl 6 (2), 21–29. 10.1016/j.atherosclerosissup.2005.02.005. 15823493

[R3] Barron A, McCarthy CM, O’Keeffe GW, 2021. Preeclampsia and neurodevelopmental outcomes: potential pathogenic roles for inflammation and oxidative stress? Mol. Neurobiol 58 (6), 2734–2756. 33492643 10.1007/s12035-021-02290-4

[R4] Bliss SL (2007). Test Reviews: Newborg, J.(2005). Battelle Developmental Inventory–Second Edition. Itasca, IL: Riverside. Journal of Psychoeducational Assessment, 25 (4), 409–415.

[R5] Bondi BC, Tassone VK, Bucsea O, Desrocher M, Pepler DJ, 2024. A Systematic Review of Neurodevelopmental Assessments in Infancy and Early Childhood: developing a Conceptual Framework, Repository of measures, and Clinical Recommendations. Neuropsychol. Rev 1–17. 10.1007/s11065-024-09641-7 38693469

[R6] Bonnin A, Levitt P, 2011. Fetal, maternal, and placental sources of serotonin and new implications for developmental programming of the brain. Neuroscience 197, 1–7. 10.1016/j.neuroscience.2011.10.005. 22001683 PMC3225275

[R7] Burd I, Bentz AI, Chai J, Gonzalez J, Monnerie H, Le Roux PD, Cohen AS, Yudkoff M, Elovitz MA, 2010. Inflammation-induced preterm birth alters neuronal morphology in the mouse fetal brain. J. Neurosci. Res 88 (9), 1872–1881. 20155801 10.1002/jnr.22368 PMC2895318

[R8] Cantonwine DE, Cordero JF, Rivera-González LO, Del Toro LVA, Ferguson KK, Mukherjee B, Calafat AM, Crespo N, Jiménez-Vélez B, Padilla IY, 2014. Urinary phthalate metabolite concentrations among pregnant women in Northern Puerto Rico: distribution, temporal variability, and predictors. Environ. Int 62, 1–11. 24161445 10.1016/j.envint.2013.09.014 PMC3874859

[R9] Carey ME, Kivumbi A, Rando J, Mesaros AC, Melnyk S, James SJ, Croen LA, Volk H, Lyall K, 2024. The association between prenatal oxidative stress levels measured by isoprostanes and offspring neurodevelopmental outcomes at 36 months. Brain, Behavior, & Immunity-Health 38, 100775. 10.1016/j.bbih.2024.100775 PMC11067487 38706573

[R10] Chatterjee S, 2016. Chapter two - Oxidative stress, Inflammation, and Disease. In: Dziubla T, Butterfield DA (Eds.), Oxidative Stress and Biomaterials. Academic Press, pp. 35–58.

[R11] Cieślik M, Gąssowska-Dobrowolska M, Jęśko H, Czapski GA, Wilkaniec A, Zawadzka A, Dominiak A, Polowy R, Filipkowski RK, Boguszewski PM, 2020. Maternal immune activation induces neuroinflammation and cortical synaptic deficits in the adolescent rat offspring. Int. J. Mol. Sci 21 (11), 4097. 32521803 10.3390/ijms21114097 PMC7312084

[R12] Clifton V, 2010. Sex and the human placenta: mediating differential strategies of fetal growth and survival. Placenta 31, S33–S39. 20004469 10.1016/j.placenta.2009.11.010

[R13] Connor CM, Dincer A, Straubhaar J, Galler JR, Houston IB, Akbarian S, 2012. Maternal immune activation alters behavior in adult offspring, with subtle changes in the cortical transcriptome and epigenome. Schizophr. Res 140 (1–3), 175–184. 22804924 10.1016/j.schres.2012.06.037 PMC3568668

[R14] Curry A, Vogel I, Skogstrand K, Drews C, Schendel D, Flanders W, Hougaard DM, Thorsen P, 2008. Maternal plasma cytokines in early-and mid-gestation of normal human pregnancy and their association with maternal factors. J. Reprod. Immunol 77 (2), 152–160. 17692390 10.1016/j.jri.2007.06.051

[R15] Dahlgren J, Samuelsson A-M, Jansson T, Holmäng A, 2006. Interleukin-6 in the maternal circulation reaches the rat fetus in mid-gestation. Pediatr. Res 60 (2), 147–151. 16864694 10.1203/01.pdr.0000230026.74139.18

[R16] Dutta S, Sengupta P, 2016. Men and mice: relating their ages. Life Sci 152, 244–248. 26596563 10.1016/j.lfs.2015.10.025

[R17] Eick SM, Barrett ES, van ‘t Erve TJ, Nguyen RH, Bush NR, Milne G, Swan SH, Ferguson KK, 2018. Association between prenatal psychological stress and oxidative stress during pregnancy. Paediatr. Perinat. Epidemiol 32 (4), 318–326. 29603338 10.1111/ppe.12465 PMC6103836

[R18] Eick SM, Ortlund K, Aguiar A, Merced-Nieves FM, Woodbury ML, Milne GL, Schantz SL, 2024. Associations between oxidative stress biomarkers during pregnancy and infant cognition at 7.5 months. Dev. Psychobiol 66 (2), e22457. 38388194 10.1002/dev.22457 PMC10901445

[R19] Elovitz MA, Brown AG, Breen K, Anton L, Maubert M, Burd I, 2011. Intrauterine inflammation, insufficient to induce parturition, still evokes fetal and neonatal brain injury. Int. J. Dev. Neurosci 29 (6), 663–671. 21382466 10.1016/j.ijdevneu.2011.02.011 PMC3140629

[R20] Ferguson KK, Rosario Z, McElrath TF, Vélez Vega C, Cordero JF, Alshawabkeh A, Meeker JD, 2019. Demographic risk factors for adverse birth outcomes in Puerto Rico in the PROTECT cohort. PLoS One 14 (6), e0217770. 31194765 10.1371/journal.pone.0217770 PMC6564423

[R21] Garay PA, Hsiao EY, Patterson PH, McAllister AK, 2013. Maternal immune activation causes age-and region-specific changes in brain cytokines in offspring throughout development. Brain Behav. Immun 31, 54–68. 22841693 10.1016/j.bbi.2012.07.008 PMC3529133

[R22] Gelman A, Hill J, 2007. Data analysis using regression and multilevel/hierarchical models. Cambridge University Press.

[R23] Ghezzi P, Floridi L, Boraschi D, Cuadrado A, Manda G, Levic S, D’Acquisto F, Hamilton A, Athersuch TJ, Selley L, 2018. Oxidative stress and inflammation induced by environmental and psychological stressors: a biomarker perspective. Antioxid. Redox Signal 28 (9), 852–872. 28494612 10.1089/ars.2017.7147

[R24] Girard S, Tremblay L, Lepage M, Sébire G, 2010. IL-1 receptor antagonist protects against placental and neurodevelopmental defects induced by maternal inflammation. J. Immunol 184 (7), 3997–4005. 20181892 10.4049/jimmunol.0903349

[R25] Goeden N, Velasquez J, Arnold KA, Chan Y, Lund BT, Anderson GM, Bonnin A, 2016. Maternal inflammation disrupts fetal neurodevelopment via increased placental output of serotonin to the fetal brain. J. Neurosci 36 (22), 6041–6049. 27251625 10.1523/JNEUROSCI.2534-15.2016 PMC4887568

[R26] Hänsel A, Hong S, Cámara RJ, Von Kaenel R, 2010. Inflammation as a psychophysiological biomarker in chronic psychosocial stress. Neurosci. Biobehav. Rev 35 (1), 115–121. 20026349 10.1016/j.neubiorev.2009.12.012

[R27] Hunter SK, Hoffman MC, D’Alessandro A, Noonan K, Wyrwa A, Freedman R, Law AJ, 2021. Male fetus susceptibility to maternal inflammation: C-reactive protein and brain development. Psychol. Med 51 (3), 450–459. 31787129 10.1017/S0033291719003313 PMC7263978

[R28] Hussain T, Murtaza G, Metwally E, Kalhoro DH, Kalhoro MS, Rahu BA, Sahito RGA, Yin Y, Yang H, Chughtai MI, 2021. The role of oxidative stress and antioxidant balance in pregnancy. Mediators Inflamm 2021 (1), 9962860. 34616234 10.1155/2021/9962860 PMC8490076

[R29] Jiménez-Osorio AS, Carreón-Torres E, Correa-Solís E, Ángel-García J, Arias-Rico J, Jiménez-Garza O, Morales-Castillejos L, Díaz-Zuleta HA, Baltazar-Tellez RM, Sánchez-Padilla ML, 2023. Inflammation and oxidative stress induced by obesity, gestational diabetes, and preeclampsia in pregnancy: role of high-density lipoproteins as vectors for bioactive compounds. Antioxidants 12 (10), 1894. 37891973 10.3390/antiox12101894 PMC10604737

[R30] Keenan-Devlin LS, Smart BP, Grobman W, Adam EK, Freedman A, Buss C, Entringer S, Miller GE, Borders AE, 2022. The intersection of race and socioeconomic status is associated with inflammation patterns during pregnancy and adverse pregnancy outcomes. Am. J. Reprod. Immunol 87 (3), e13489. 34958140 10.1111/aji.13489

[R31] Kelly RS, Lee-Sarwar K, Chen Y-C, Laranjo N, Fichorova R, Chu SH, Prince N, Lasky-Su J, Weiss ST, Litonjua AA, 2022. Maternal inflammatory biomarkers during pregnancy and early life neurodevelopment in offspring: results from the VDAART study. Int. J. Mol. Sci 23 (23), 15249. 36499584 10.3390/ijms232315249 PMC9739845

[R32] Kim C, Cathey AL, Watkins DJ, Mukherjee B, Rosario-Pabón ZY, Vélez-Vega CM, Alshawabkeh AN, Cordero JF, Meeker JD, 2022. Maternal blood metal concentrations are associated with matrix metalloproteinases (MMPs) among pregnant women in Puerto Rico. Environ. Res 209, 112874. 10.1016/j.envres.2022.112874. 35123972 PMC10443181

[R33] Lagranha CJ, Silva TLA, Silva SCA, Braz GRF, da Silva AI, Fernandes MP, Sellitti DF, 2018. Protective effects of estrogen against cardiovascular disease mediated via oxidative stress in the brain. Life Sci 192, 190–198. 29191645 10.1016/j.lfs.2017.11.043

[R34] Lee AC, Cherkerzian S, Tofail F, Folger LV, Ahmed S, Rahman S, Chowdhury NH, Khanam R, Olson I, Oken E, 2024. Perinatal inflammation, fetal growth restriction, and long-term neurodevelopmental impairment in Bangladesh. Pediatr. Res 1–11. 10.1038/s41390-024-03101-x PMC11959561 38589559

[R35] Lee CW, Cathey AL, Watkins DJ, Rosario-Pabón ZY, Vélez-Vega CM, Alshawabkeh AN, Cordero JF, Meeker JD, 2023. Associations of urinary phthalate metabolites and inflammatory biomarkers among pregnant women in Puerto Rico. Sci. Total Environ 854, 158773. 36113809 10.1016/j.scitotenv.2022.158773 PMC10323976

[R36] Lee HS, Kim WJ, 2022. The Role of Matrix Metalloproteinase in Inflammation with a Focus on Infectious Diseases. Int. J. Mol. Sci 23 (18). 10.3390/ijms231810546. PMC9500641 36142454

[R37] Lenroot RK, Giedd JN, 2006. Brain development in children and adolescents: insights from anatomical magnetic resonance imaging. Neurosci. Biobehav. Rev 30 (6), 718–729. 16887188 10.1016/j.neubiorev.2006.06.001

[R38] Linderkamp O, Janus L, Linder R, Skoruppa DB, 2009. Time table of normal foetal brain development. International Journal of Prenatal and Perinatal Psychology and Medicine 21 (1/2), 4–16.

[R39] Lugrin J, Rosenblatt-Velin N, Parapanov R, Liaudet L, 2014. The role of oxidative stress during inflammatory processes. Biol. Chem 395 (2), 203–230. 24127541 10.1515/hsz-2013-0241

[R40] Lushchak VI, 2014. Free radicals, reactive oxygen species, oxidative stress and its classification. Chem. Biol. Interact 224, 164–175. 25452175 10.1016/j.cbi.2014.10.016

[R41] Manjourides J, Zimmerman E, Watkins DJ, Carpenito T, Vélez-Vega CM, Huerta-Montañez G, Rosario Z, Ayala I, Vergara C, Feric Z, 2020. Cohort profile: Center for research on early childhood exposure and development in Puerto Rico. BMJ Open 10 (7), e036389. 10.1136/bmjopen-2019-036389 PMC7371225 32690520

[R42] McEwan F, Glazier JD, Hager R, 2023. The impact of maternal immune activation on embryonic brain development. Front. Neurosci 17, 1146710. 36950133 10.3389/fnins.2023.1146710 PMC10025352

[R43] McNearney TA, Westlund KN, 2023. Pluripotential GluN1 (NMDA NR1): Functional significance in Cellular Nuclei in Pain/Nociception. Int. J. Mol. Sci 24 (17). 10.3390/ijms241713196. PMC10488196 37686003

[R44] Meeker JD, Cantonwine DE, Rivera-González LO, Ferguson KK, Mukherjee B, Calafat AM, Ye X, Anzalota Del Toro LV, Crespo-Hernández N, Jiménez-Vélez B, 2013. Distribution, variability, and predictors of urinary concentrations of phenols and parabens among pregnant women in Puerto Rico. Environ. Sci. Technol 47 (7), 3439–3447. 23469879 10.1021/es400510g PMC3638245

[R45] Meeker JD, Hu H, Cantonwine DE, Lamadrid-Figueroa H, Calafat AM, Ettinger AS, Hernandez-Avila M, Loch-Caruso R, Téllez-Rojo MM, 2009. Urinary phthalate metabolites in relation to preterm birth in Mexico City. Environ. Health Perspect 117 (10), 1587–1592. 20019910 10.1289/ehp.0800522 PMC2790514

[R46] Minakova E, Warner BB, 2018. Maternal immune activation, central nervous system development and behavioral phenotypes. Birth Defects Res 110 (20), 1539–1550. 30430765 10.1002/bdr2.1416

[R47] Mocayar Marón FJ, Ferder L, Saraví FD, Manucha W, 2019. Hypertension linked to allostatic load: from psychosocial stress to inflammation and mitochondrial dysfunction. Stress 22 (2), 169–181. 30547701 10.1080/10253890.2018.1542683

[R48] Mor G, 2008. Inflammation and pregnancy: the role of toll-like receptors in trophoblast–immune interaction. Ann. N. Y. Acad. Sci 1127 (1), 121–128. 18443339 10.1196/annals.1434.006

[R49] Morgan JE, Lee SS, Mahrer NE, Guardino CM, Davis EP, Shalowitz MU, Ramey SL, Dunkel Schetter C, 2020. Prenatal maternal C-reactive protein prospectively predicts child executive functioning at ages 4–6 years. Dev. Psychobiol 62 (8), 1111–1123. 32441781 10.1002/dev.21982 PMC7680271

[R50] Münzel T, Daiber A, 2018. Environmental stressors and their impact on health and disease with focus on oxidative stress. Antioxid. Redox Signal 28 (9), 735–740. 29278923 10.1089/ars.2017.7488

[R51] Nazzari S, Fearon P, Rice F, Ciceri F, Molteni M, Frigerio A, 2020. Neuroendocrine and immune markers of maternal stress during pregnancy and infant cognitive development. Dev. Psychobiol 62 (8), 1100–1110. 32232990 10.1002/dev.21967

[R52] Oldenburg KS, O’Shea TM, Fry RC, 2020. Genetic and epigenetic factors and early life inflammation as predictors of neurodevelopmental outcomes. Semin. Fetal Neonatal Med10.1016/j.siny.2020.101115 PMC7363586 32444251

[R53] Osman HC, Moreno R, Rose D, Rowland ME, Ciernia AV, Ashwood P, 2024. Impact of maternal immune activation and sex on placental and fetal brain cytokine and gene expression profiles in a preclinical model of neurodevelopmental disorders. J. Neuroinflammation 21 (1), 118. 38715090 10.1186/s12974-024-03106-7 PMC11077729

[R54] Prokai L, Prokai-Tatrai K, Perjési P, Simpkins JW, 2005. Mechanistic insights into the direct antioxidant effects of estrogens. Drug Dev. Res 66 (2), 118–125.

[R55] Ramiro-Cortijo D, Rodríguez-Rodríguez P, Lopez De Pablo Á, López-Giménez MR, González MC, & Arribas SM (2017). Fetal undernutrition and oxidative stress: Influence of sex and gender. Handbook of Famine, Starvation, and Nutrient Deprivation: From Biology to Policy; Preedy VR, Patel VB, Eds.

[R56] Rana SA, Aavani T, Pittman QJ, 2012. Sex effects on neurodevelopmental outcomes of innate immune activation during prenatal and neonatal life. Horm. Behav 62 (3), 228–236. 10.1016/j.yhbeh.2012.03.015. 22516179 PMC3522744

[R57] Rees S, Harding R, 2004. Brain development during fetal life: influences of the intrauterine environment. Neurosci. Lett 361 (1–3), 111–114. 15135906 10.1016/j.neulet.2004.02.002

[R58] Reyes MR, Sifuentes-Alvarez A, Lazalde B, 2006. Estrogens are potentially the only steroids with an antioxidant role in pregnancy: in vitro evidence. Acta Obstet. Gynecol. Scand 85 (9), 1090–1093. 16929413 10.1080/00016340500453685

[R59] Rommel A-S, Milne GL, Barrett ES, Bush NR, Nguyen R, Sathyanarayana S, Swan SH, Ferguson KK, 2020. Associations between urinary biomarkers of oxidative stress in the third trimester of pregnancy and behavioral outcomes in the child at 4 years of age. Brain Behav. Immun 90, 272–278. 32905853 10.1016/j.bbi.2020.08.029 PMC7544682

[R60] Sal-Sarria S, Conejo NM, Gonźalez-Pardo H, 2024. Maternal Immune Activation and its Multifaceted Effects on Learning and memory in Rodent Offspring: a Systematic Review. Neurosci. Biobehav. Rev 105844. 10.1016/j.neubiorev.2024.105844 39106940

[R61] Scott H, Phillips TJ, Stuart GC, Rogers MF, Steinkraus BR, Grant S, Case CP, 2018. Preeclamptic placentae release factors that damage neurons: implications for foetal programming of disease. Neuronal Signaling 2 (4), NS20180139. 10.1042/NS20180139 PMC7363326 32714596

[R62] Singh V, Kaur R, Kumari P, Pasricha C, Singh R, 2023. ICAM-1 and VCAM-1: Gatekeepers in various inflammatory and cardiovascular disorders. Clin. Chim. Acta 117487. 10.1016/j.cca.2023.117487 37442359

[R63] Stark MJ, Hodyl NA, Wright IM, Clifton VL, 2011. Influence of sex and glucocorticoid exposure on preterm placental pro-oxidant-antioxidant balance. Placenta 32 (11), 865–870. 21903264 10.1016/j.placenta.2011.08.010

[R64] Van’t Erve TJ, Lih FB, Jelsema C, Deterding LJ, Eling TE, Mason RP, Kadiiska MB, 2016. Reinterpreting the best biomarker of oxidative stress: the 8-iso-prostaglandin F2α/prostaglandin F2α ratio shows complex origins of lipid peroxidation biomarkers in animal models. Free Radic. Biol. Med 95, 65–73. 26964509 10.1016/j.freeradbiomed.2016.03.001 PMC6626672

[R65] Wadley AJ, Veldhuijzen van Zanten JJ, Aldred S, 2013. The interactions of oxidative stress and inflammation with vascular dysfunction in ageing: the vascular health triad. Age 35, 705–718. 22453933 10.1007/s11357-012-9402-1 PMC3636404

[R66] Wiley RW, Rapp B, 2019. Statistical analysis in Small-N designs: using linear mixed-effects modeling for evaluating intervention effectiveness. Aphasiology 33 (1), 1–30. 10.1080/02687038.2018.1454884. 33012945 PMC7531584

[R67] Zaretsky MV, Alexander JM, Byrd W, Bawdon RE, 2004. Transfer of inflammatory cytokines across the placenta. Obstet. Gynecol 103 (3), 546–550. 14990420 10.1097/01.AOG.0000114980.40445.83

[R68] Zhang Z, 2016. Multiple imputation with multivariate imputation by chained equation (MICE) package. Annals of Translational Medicine 4 (2), 30. 26889483 10.3978/j.issn.2305-5839.2015.12.63 PMC4731595

