# Supplementary material for: Maternal inflammation and oxidative stress during pregnancy in relation to early childhood neurodevelopment
Source: Brain Behav Immun. Author manuscript; Available in PMC 2026 Jun 26. (PMC13307287; doi:10.1016/j.bbi.2026.106514)
Supplement: 1 [file NIHMS2189094-supplement-1.docx]

**Supplementary Materials**

**Supplementary Table S1.** Distribution of gestational age (week) at each study visit for inflammation biomarker concentration (ng/mL) and oxidative stress biomarker. Q25: 25^th^ percentile. Q75: 75^th^ percentile.

|  |  | Gestational age (week) | | | | | |
| --- | --- | --- | --- | --- | --- | --- | --- |
| Measurement | Gestational Visit | Mean | Median | Q25 | Q75 | Minimum | Maximum |
| Inflammation marker | 1 | 18 | 18 | 16 | 19 | 9 | 28 |
|  | 3 | 28 | 27 | 26 | 29 | 17 | 40 |
| Oxidative stress marker | 1 | 18 | 18 | 17 | 19 | 8 | 30 |
|  | 2 | 23 | 23 | 21 | 25 | 13 | 37 |
|  | 3 | 28 | 27 | 26 | 30 | 13 | 41 |

**Supplementary Table S2**. Distributions of inflammation biomarker concentration (ng/mL) in the study participants, by potential confounders (categorical). GM: geometric mean. IQR: interquartile range. p-values were calculated using the Wilcoxon Rank Sum test for two-group comparisons and the Kruskal-Wallis test for multi-group comparisons. These non-parametric tests compare differences in medians.

Bold text of p-value indicates statistical significance (p-value < 0.05).

Note: ^1^ Total number of observations by child age was 272 for child age groups.

|  | GM (IQR) | | | | | |
| --- | --- | --- | --- | --- | --- | --- |
| Category | CRP | ICAM | VCAM | MMP1 | MMP2 | MMP9 |
| **Maternal Age** |  |  |  |  |  |  |
| **p-value** | 0.124 | **0.007** | **0.043** | 0.139 | 0.514 | 0.14 |
| 18-24 | 2745.09 (1626.8, 5972.11) | 771.77 (579.85, 867.64) | 261.94 (220.41, 318.06) | 0.25 (0.16, 0.54) | 10.28 (7.4, 10.07) | 35.28 (28.84, 48.4) |
| 25-29 | 2835.85 (1306.25, 6445.17) | 624.08 (489.57, 705.25) | 221.05 (187.76, 287.52) | 0.27 (0.14, 0.46) | 10.73 (7.76, 10.69) | 31.03 (22.6, 47.16) |
| 30-34 | 2183.65 (1254.39, 4452.68) | 595.25 (455.82, 704.41) | 207.88 (172.07, 276.08) | 0.37 (0.24, 0.62) | 10.48 (8.23, 11.09) | 28.54 (22.14, 42.24) |
| 35-41 | 3512.23 (1684.91, 6689.75) | 579.46 (516.91, 662.01) | 236.01 (206.35, 300.98) | 0.28 (0.16, 0.5) | 9.58 (8.05, 9.57) | 27.99 (19.05, 42.29) |
| **Maternal Education** |  |  |  |  |  |  |
| **p-value** | 0.526 | **0.03** | 0.055 | 0.748 | 0.065 | 0.719 |
| GED or less | 2854.71 (1636.11, 6141.52) | 780.29 (573.47, 928.5) | 263.9 (229.07, 315.84) | 0.28 (0.16, 0.55) | 11.46 (8.65, 10.71) | 30.29 (20.04, 43.05) |
| Some college | 2958.25 (1529.65, 6451.2) | 660.31 (508.97, 699.27) | 241.19 (195.08, 312.58) | 0.26 (0.18, 0.46) | 9.92 (7.35, 10.42) | 32.8 (25.56, 48.95) |
| Bachelors or higher | 2519.18 (1263.98, 5261.9) | 600.83 (473.9, 721.58) | 212.69 (180.52, 284.64) | 0.31 (0.18, 0.58) | 10.44 (8.2, 10.69) | 29.75 (21.82, 45.06) |
| **Current Job** |  |  |  |  |  |  |
| **p-value** | 0.665 | **0.014** | 0.192 | 0.536 | 0.663 | 0.068 |
| No | 2947.47 (1522.88, 6509.3) | 740.18 (550.25, 880.27) | 249.2 (209.67, 307.26) | 0.26 (0.15, 0.53) | 10.85 (7.78, 10.71) | 34.64 (26.9, 51.9) |
| Yes | 2659.83 (1291.2, 5495.49) | 608.46 (489.45, 705.4) | 222.71 (186.82, 299.15) | 0.29 (0.18, 0.54) | 10.19 (7.79, 10.7) | 29.45 (21.88, 43.36) |
| **Pre-pregnancy BMI** |  |  |  |  |  |  |
| **p-value** | **0.001** | 0.305 | 0.063 | 0.479 | **0.029** | 0.738 |
| (0, 25] | 2060.44 (1264.99, 4506.42) | 625.79 (482.45, 725.72) | 213.23 (185.74, 284.67) | 0.26 (0.16, 0.48) | 11.85 (8.26, 11.51) | 29.46 (21.84, 45.56) |
| (25, 29.9] | 3214.78 (1467.74, 6707.9) | 685.69 (549, 778.07) | 234.59 (183.49, 308.21) | 0.3 (0.24, 0.56) | 9.14 (7.77, 9.94) | 32.14 (23.03, 49.19) |
| (29.9, 51] | 4233.4 (2510.44, 8379.28) | 629.14 (516.99, 760.52) | 258.18 (217.49, 355.83) | 0.3 (0.15, 0.55) | 9.66 (7.32, 10.09) | 31.57 (22.45, 42.62) |
| **Marital Status** |  |  |  |  |  |  |
| **p-value** | 0.443 | 0.405 | 0.565 | 0.561 | 0.257 | 0.495 |
| Single | 3120.45 (1392.6, 7428.35) | 585.19 (500.21, 650.76) | 251.35 (212.74, 295.88) | 0.25 (0.15, 0.46) | 9.6 (7.32, 9.6) | 33.98 (26.46, 46.58) |
| Married | 2814.06 (1365.99, 5286.32) | 661.19 (507.47, 758.51) | 235.52 (197.82, 289.01) | 0.3 (0.18, 0.54) | 10.32 (8.01, 10.7) | 31.28 (22.97, 46.81) |
| Cohabitating | 2436.84 (1210.56, 6018.45) | 632.04 (463.6, 758.41) | 213.45 (154.85, 310.36) | 0.27 (0.14, 0.52) | 10.54 (7.83, 11.32) | 28.66 (21.83, 41.61) |
| **Household Income** |  |  |  |  |  |  |
| **p-value** | 0.339 | 0.196 | 0.113 | 0.515 | 0.076 | 0.187 |
| <10k | 2688.21 (1523.7, 6111.21) | 751.56 (508.97, 872.3) | 253.24 (232.83, 305.97) | 0.33 (0.19, 0.56) | 11.05 (8.24, 10.94) | 32.44 (25.56, 48.75) |
| 10k - <30k | 2710.93 (1347.9, 5074.91) | 604.5 (539.54, 717.5) | 227.83 (186.38, 308.54) | 0.25 (0.17, 0.43) | 9.22 (7.43, 10.4) | 33.09 (25.3, 45.59) |
| 30k - <50k | 3063.18 (1627.51, 5991.58) | 626.71 (464.49, 713.38) | 212.19 (170.22, 279.65) | 0.31 (0.16, 0.58) | 10.37 (7.79, 10.02) | 27.27 (21.46, 40.96) |
| ≥50k | 1868.66 (1016.36, 4355.52) | 557.72 (469.77, 651.35) | 211.55 (188.4, 271.07) | 0.27 (0.15, 0.53) | 13.38 (8.82, 12.11) | 29.68 (15.85, 46.18) |
| **Alcohol Use** |  |  |  |  |  |  |
| **p-value** | 0.882 | 0.118 | 0.86 | 0.074 | 0.179 | 0.175 |
| Never | 2809.51 (1447.11, 6162.43) | 662.94 (514.2, 720.33) | 230.9 (195.08, 295.21) | 0.3 (0.18, 0.58) | 11.41 (8.2, 11.2) | 32.83 (25.56, 51.39) |
| Yes, before pregnancy | 2606.48 (1246.53, 5044.73) | 629.1 (469.77, 754.96) | 226.51 (189.33, 309.5) | 0.27 (0.15, 0.44) | 9.43 (7.71, 10.45) | 29.28 (21.8, 41.94) |
| Yes, currently | 2575.53 (1647.59, 4358.5) | 512.31 (482.49, 591.21) | 225.56 (183.19, 263.29) | 0.29 (0.26, 0.87) | 10.97 (8.25, 10.73) | 26.29 (22.6, 41.97) |
| **Pregnancy Number** |  |  |  |  |  |  |
| **p-value** | 0.596 | 0.722 | 0.182 | 0.728 | 0.072 | 0.163 |
| 0 | 2696.03 (1331.87, 6504.77) | 599.77 (473.17, 721.58) | 217.01 (157.11, 305.97) | 0.27 (0.18, 0.52) | 10.52 (7.73, 10.72) | 32.53 (26.79, 45.19) |
| 1 | 2589.23 (1300.32, 4946.07) | 674.71 (501.61, 773.41) | 235.58 (196.2, 262) | 0.32 (0.23, 0.48) | 9.69 (7.76, 9.92) | 31.93 (24.28, 49.51) |
| 2 - 5 | 2970.59 (1978.71, 5858.99) | 671.79 (521.07, 734.38) | 242.33 (224.15, 307.96) | 0.27 (0.13, 0.59) | 11.11 (8.21, 11.18) | 26.61 (17.7, 44.27) |
| **Child Sex** |  |  |  |  |  |  |
| **p-value** | 0.374 | 0.99 | 0.393 | 0.422 | 0.302 | 0.945 |
| Female | 2819.47 (1447.11, 6451.2) | 635.11 (519.78, 755.84) | 240.14 (203.79, 301.34) | 0.27 (0.15, 0.53) | 10.28 (7.72, 10.33) | 30.58 (21.66, 48.13) |
| Male | 2605.99 (1319.06, 4966.02) | 647.99 (474.92, 742.44) | 219.74 (183.47, 302.98) | 0.3 (0.19, 0.55) | 10.48 (8.03, 10.96) | 31.05 (23.42, 44.77) |
| **Child Age Group^1^** |  |  |  |  |  |  |
| **p-value** | 0.284 | 0.577 | **0.017** | **0.021** | 0.109 | 0.084 |
| 1 year (N=104) | 3122.99 (1523.7, 6726.05) | 654.28 (524.84, 760.98) | 214.87 (185.14, 285.31) | 0.34 (0.19, 0.65) | 11.61 (7.78, 11.13) | 32.28 (25.59, 47.16) |
| 2 years (N=81) | 2737.62 (1520.4, 5449.97) | 668.95 (508.97, 791.76) | 256.73 (213.74, 313.65) | 0.26 (0.16, 0.46) | 8.58 (7.66, 10.21) | 26.82 (17.75, 44.48) |
| 3 years (N=81) | 2518.55 (1238.48, 4946.07) | 647.19 (479.08, 718.81) | 241.62 (192.49, 297.23) | 0.25 (0.14, 0.45) | 9.76 (8.35, 10.72) | 31.85 (22.77, 43.1) |

**Supplementary Table S3**. Distributions of specific gravity adjusted oxidative stress biomarker concentration (ng/mL) in the study participants, by potential confounders (categorical). GM: geometric mean. IQR: interquartile range. p-values were calculated using the Wilcoxon Rank Sum test for two-group comparisons and the Kruskal-Wallis test for multi-group comparisons. These non-parametric tests compare differences in medians. Bold text of p-value indicates statistical significance (p-value < 0.05).

Note: ^1^ Total number of observations by child age was 272 for child age groups.

| Category | PGF2a | IsoP | fPGHS | fCLP | aPGHS | aCLP |
| --- | --- | --- | --- | --- | --- | --- |
| **Maternal Age** |  |  |  |  |  |  |
| **p-value** | **<0.01** | **0.01** | 0.9 | 0.36 | 0.34 | 0.14 |
| 18-24 | 3.7 (2.75, 4.87) | 1.83 (1.3, 2.55) | 0.11 (0.04, 0.51) | 0.63 (0.51, 0.86) | 0.19 (0.08, 0.62) | 1.11 (0.83, 2.03) |
| 25-29 | 2.78 (2.05, 3.92) | 1.48 (1.14, 2.06) | 0.1 (0.02, 0.44) | 0.71 (0.49, 0.95) | 0.12 (0.03, 0.46) | 0.91 (0.65, 1.66) |
| 30-34 | 3.01 (2.21, 3.89) | 1.59 (1.18, 2.26) | 0.11 (0.03, 0.38) | 0.73 (0.56, 0.94) | 0.16 (0.07, 0.45) | 1.04 (0.72, 1.76) |
| 35-41 | 2.88 (1.92, 4.17) | 1.4 (1, 2.12) | 0.13 (0.04, 0.53) | 0.68 (0.5, 0.91) | 0.16 (0.05, 0.49) | 0.83 (0.59, 1.71) |
| **Maternal Education** |  |  |  |  |  |  |
| **p-value** | 0.07 | **<0.01** | 0.19 | 0.92 | 0.29 | **0.02** |
| GED or less | 3.52 (2.53, 4.91) | 1.91 (1.39, 3.02) | 0.06 (0.01, 0.37) | 0.65 (0.55, 0.95) | 0.11 (0.02, 0.49) | 1.19 (0.93, 2.22) |
| Some college | 3.17 (2.18, 4.48) | 1.57 (1.18, 2.32) | 0.14 (0.06, 0.55) | 0.7 (0.52, 0.91) | 0.19 (0.09, 0.55) | 0.97 (0.73, 1.83) |
| Bachelors or higher | 2.93 (2.11, 4.04) | 1.52 (1.12, 2.18) | 0.11 (0.02, 0.45) | 0.69 (0.5, 0.92) | 0.15 (0.05, 0.47) | 0.94 (0.64, 1.68) |
| **Current Job** |  |  |  |  |  |  |
| **p-value** | 0.3 | **<0.01** | 0.11 | 0.57 | 0.92 | **0.02** |
| No | 3.25 (2.32, 4.11) | 1.82 (1.29, 2.52) | 0.08 (0.02, 0.37) | 0.67 (0.51, 0.89) | 0.14 (0.05, 0.54) | 1.2 (0.83, 2.09) |
| Yes | 3 (2.17, 4.19) | 1.49 (1.14, 2.21) | 0.13 (0.04, 0.47) | 0.7 (0.5, 0.94) | 0.16 (0.05, 0.47) | 0.9 (0.64, 1.74) |
| **Pre-pregnancy BMI** |  |  |  |  |  |  |
| **p-value** | **0.01** | **<0.01** | 0.19 | **0.03** | 0.15 | **0.01** |
| (0, 25] | 2.81 (1.93, 4.06) | 1.39 (1.09, 1.98) | 0.12 (0.03, 0.56) | 0.72 (0.57, 0.99) | 0.14 (0.05, 0.47) | 0.85 (0.64, 1.57) |
| (25, 29.9] | 3.19 (2.46, 4.02) | 1.78 (1.31, 2.38) | 0.09 (0.02, 0.34) | 0.7 (0.58, 0.92) | 0.15 (0.06, 0.46) | 1.18 (0.72, 1.93) |
| (29.9, 51] | 3.58 (2.36, 5.36) | 1.89 (1.36, 2.43) | 0.12 (0.06, 0.37) | 0.62 (0.48, 0.8) | 0.22 (0.1, 0.67) | 1.13 (0.83, 1.89) |
| **Marital Status** |  |  |  |  |  |  |
| **p-value** | 0.8 | 0.3 | 0.27 | 0.19 | 0.52 | 0.29 |
| Single | 2.94 (2.28, 3.73) | 1.68 (1.11, 2.38) | 0.07 (0.05, 0.29) | 0.71 (0.6, 0.91) | 0.11 (0.11, 0.42) | 1.14 (0.64, 1.97) |
| Married | 3.11 (2.18, 4.31) | 1.64 (1.25, 2.34) | 0.11 (0.02, 0.45) | 0.71 (0.51, 0.94) | 0.15 (0.05, 0.53) | 1.02 (0.76, 1.81) |
| Cohabitating | 3.09 (2.12, 4.35) | 1.44 (1.08, 2.24) | 0.13 (0.04, 0.5) | 0.63 (0.48, 0.89) | 0.18 (0.07, 0.47) | 0.84 (0.64, 1.68) |
| **Household Income** |  |  |  |  |  |  |
| **p-value** | 0.2 | 0.46 | 0.08 | 0.91 | 0.49 | 0.41 |
| <10k | 3.08 (2.26, 3.91) | 1.7 (1.18, 2.4) | 0.07 (0.02, 0.37) | 0.67 (0.6, 0.88) | 0.13 (0.04, 0.46) | 1.14 (0.79, 2.09) |
| 10k - <30k | 3.07 (2.37, 4.18) | 1.53 (1.2, 2.28) | 0.14 (0.06, 0.47) | 0.71 (0.54, 0.98) | 0.18 (0.11, 0.46) | 0.91 (0.7, 1.76) |
| 30k - <50k | 2.74 (1.91, 3.92) | 1.59 (1.23, 2.21) | 0.08 (0.02, 0.32) | 0.73 (0.57, 0.94) | 0.12 (0.03, 0.45) | 1.07 (0.74, 1.72) |
| ≥50k | 2.93 (2.17, 4.06) | 1.48 (1.02, 2.01) | 0.13 (0.02, 0.57) | 0.75 (0.5, 1) | 0.15 (0.05, 0.53) | 0.89 (0.49, 1.91) |
| **Alcohol Use** |  |  |  |  |  |  |
| **p-value** | 0.16 | 0.35 | 0.54 | 0.12 | 0.42 | 0.18 |
| Never | 2.95 (2.08, 3.92) | 1.6 (1.22, 2.27) | 0.1 (0.03, 0.44) | 0.71 (0.57, 0.95) | 0.14 (0.05, 0.47) | 1.01 (0.72, 1.76) |
| Yes, before pregnancy | 3.19 (2.39, 4.6) | 1.58 (1.18, 2.36) | 0.12 (0.04, 0.47) | 0.68 (0.51, 0.88) | 0.17 (0.07, 0.52) | 0.97 (0.7, 1.86) |
| Yes, currently | 3.58 (2.32, 4.36) | 1.3 (0.99, 1.48) | 0.26 (0.21, 0.67) | 0.5 (0.28, 0.91) | 0.32 (0.21, 0.38) | 0.63 (0.34, 1.09) |
| **Pregnancy Number** |  |  |  |  |  |  |
| **p-value** | 0.51 | 0.27 | 0.31 | 0.2 | 0.99 | 0.36 |
| 0 | 3.03 (2.37, 4.19) | 1.54 (1.25, 2.25) | 0.12 (0.03, 0.51) | 0.72 (0.58, 0.95) | 0.15 (0.07, 0.52) | 0.94 (0.78, 1.81) |
| 1 | 3.26 (2.23, 4.56) | 1.72 (1.18, 2.45) | 0.09 (0.02, 0.35) | 0.64 (0.49, 0.89) | 0.15 (0.05, 0.46) | 1.09 (0.64, 2.12) |
| 2-5 | 2.94 (2.08, 4.06) | 1.48 (1.15, 2.09) | 0.12 (0.04, 0.53) | 0.7 (0.5, 0.94) | 0.16 (0.08, 0.52) | 0.91 (0.72, 1.57) |
| **Child Sex** |  |  |  |  |  |  |
| **p-value** | 0.1 | 0.96 | 0.25 | 0.19 | 0.05 | 0.7 |
| Female | 2.95 (2.18, 3.73) | 1.57 (1.15, 2.31) | 0.09 (0.02, 0.43) | 0.72 (0.57, 0.93) | 0.13 (0.04, 0.46) | 1 (0.7, 1.91) |
| Male | 3.23 (2.22, 4.64) | 1.6 (1.18, 2.38) | 0.13 (0.05, 0.48) | 0.65 (0.5, 0.9) | 0.19 (0.08, 0.55) | 0.96 (0.69, 1.67) |
| **Child age** |  |  |  |  |  |  |
| **p-value** | **<0.01** | 0.35 | **<0.01** | **0.01** | **<0.01** | **0.04** |
| 1 year (N=123) | 2.82 (1.93, 4.02) | 1.5 (1.18, 2.27) | 0.09 (0.02, 0.37) | 0.7 (0.56, 0.92) | 0.12 (0.04, 0.43) | 0.93 (0.74, 1.85) |
| 2 years (N=114) | 3.56 (2.47, 4.8) | 1.57 (1.06, 2.33) | 0.16 (0.06, 0.61) | 0.62 (0.41, 0.88) | 0.23 (0.11, 0.6) | 0.87 (0.48, 1.67) |
| 3 years (N=84) | 2.88 (2.18, 3.86) | 1.75 (1.25, 2.39) | 0.09 (0.02, 0.35) | 0.78 (0.6, 0.96) | 0.14 (0.05, 0.46) | 1.26 (0.9, 1.91) |

**Supplementary Table S4**. Number of participants with BDI-2 domain scores, repeated measurements, and distribution of BDI-2 domain scores by age group. BDI-2 domain scores were measured at approximately 1 and 2 years of age, with an additional Motor-domain assessment at 3 years.

| 4Inflammation analyses sample | | | | | | | | | | | |
| --- | --- | --- | --- | --- | --- | --- | --- | --- | --- | --- | --- |
| Age group | BDI Domain | | Numbers of participants (Observations) | Numbers of participants | Numbers of repeated participants | Mean | Median | Q25 | Q75 | Min | Max |
| 1 year | Adaptive | | 142 (171) | 98 | 29 | 102.2 | 105 | 100 | 110 | 55 | 125 |
| 2 years | Adaptive | |  | 73 |  | 97.2 | 100 | 94 | 105 | 55 | 120 |
| 1 year | Cognitive | | 137 (163) | 98 | 26 | 100.5 | 100 | 97 | 107 | 67 | 117 |
| 2 years | Cognitive | |  | 65 |  | 87 | 88.5 | 83 | 93 | 56 | 102 |
| 1 year | Communication | | 143 (173) | 98 | 30 | 99.1 | 100 | 95 | 108 | 62 | 127 |
| 2 years | Communication | |  | 75 |  | 92.3 | 95 | 80 | 108 | 55 | 138 |
| 1 year | Motor | | 172 (234) | 98 | 1 year & 2 years=28;  2 years & 3 years=23;  1 year & 3 years=21;  All three ages=10 | 104 | 104 | 100 | 110 | 70 | 125 |
| 2 years | Motor | |  | 71 |  | 101.8 | 103 | 98 | 107 | 70 | 113 |
| 3 years | Motor | |  | 65 |  | 100 | 100 | 93 | 105 | 72 | 125 |
| 1 year | Personal-Social | | 140 (168) | 96 | 28 | 105.1 | 105 | 100 | 110 | 80 | 130 |
| 2 years | Personal-Social | |  | 72 |  | 102.6 | 105 | 98 | 109.5 | 60 | 125 |
| 1 year | Total | | 132 (154) | 96 | 22 | 102.8 | 104 | 99 | 108.8 | 73 | 126 |
| 2 years | Total | |  | 58 |  | 96.4 | 98 | 92.5 | 103.5 | 56 | 113 |
| Oxidative stress analyses sample | | | | | | | | | | | |
| Age group | | BDI Domain | Numbers of participants (Observations) | Numbers of participants | Numbers of repeated participants | Mean | Median | Q25 | Q75 | Min | Max |
| 1 year | | Adaptive | 187 (218) | 112 | 31 | 104 | 105 | 100 | 110 | 70 | 125 |
| 2 years | | Adaptive |  | 106 |  | 97.5 | 100 | 92.2 | 105 | 55 | 140 |
| 1 year | | Cognitive | 176 (204) | 112 | 28 | 101.7 | 103 | 100 | 107 | 67 | 117 |
| 2 years | | Cognitive |  | 92 |  | 87.4 | 89 | 83 | 93 | 56 | 103 |
| 1 year | | Communication | 188 (220) | 112 | 32 | 100.3 | 100 | 95 | 108 | 67 | 127 |
| 2 years | | Communication |  | 108 |  | 91.8 | 93 | 81 | 105 | 55 | 138 |
| 1 year | | Motor | 217 (283) | 112 | 1 year & 2 years=30;  2 years & 3 years=24;  1 year & 3 years=22;  All three ages=10 | 104.8 | 104 | 100 | 110 | 88 | 125 |
| 2 years | | Motor |  | 102 |  | 103.1 | 104 | 100 | 108 | 70 | 116 |
| 3 years | | Motor |  | 69 |  | 100 | 100 | 93 | 105 | 72 | 125 |
| 1 year | | Personal-Social | 182 (212) | 111 | 30 | 106.4 | 105 | 103 | 110 | 88 | 133 |
| 2 years | | Personal-Social |  | 101 |  | 103.2 | 105 | 98 | 110 | 60 | 135 |
| 1 year | | Total | 167 (191) | 111 | 24 | 104.1 | 104 | 99 | 109 | 88 | 126 |
| 2 years | | Total |  | 80 |  | 96.4 | 98 | 91 | 104 | 56 | 129 |

**Supplementary Figure S1.** Spearman correlations of biomarker concentrations across gestational visits for (a) inflammation markers and (b) oxidative stress markers. Samples were collected at approximately 18 weeks (visit 1), 23 weeks (visit 2), and 27 weeks (visit 3) of gestation.

a.


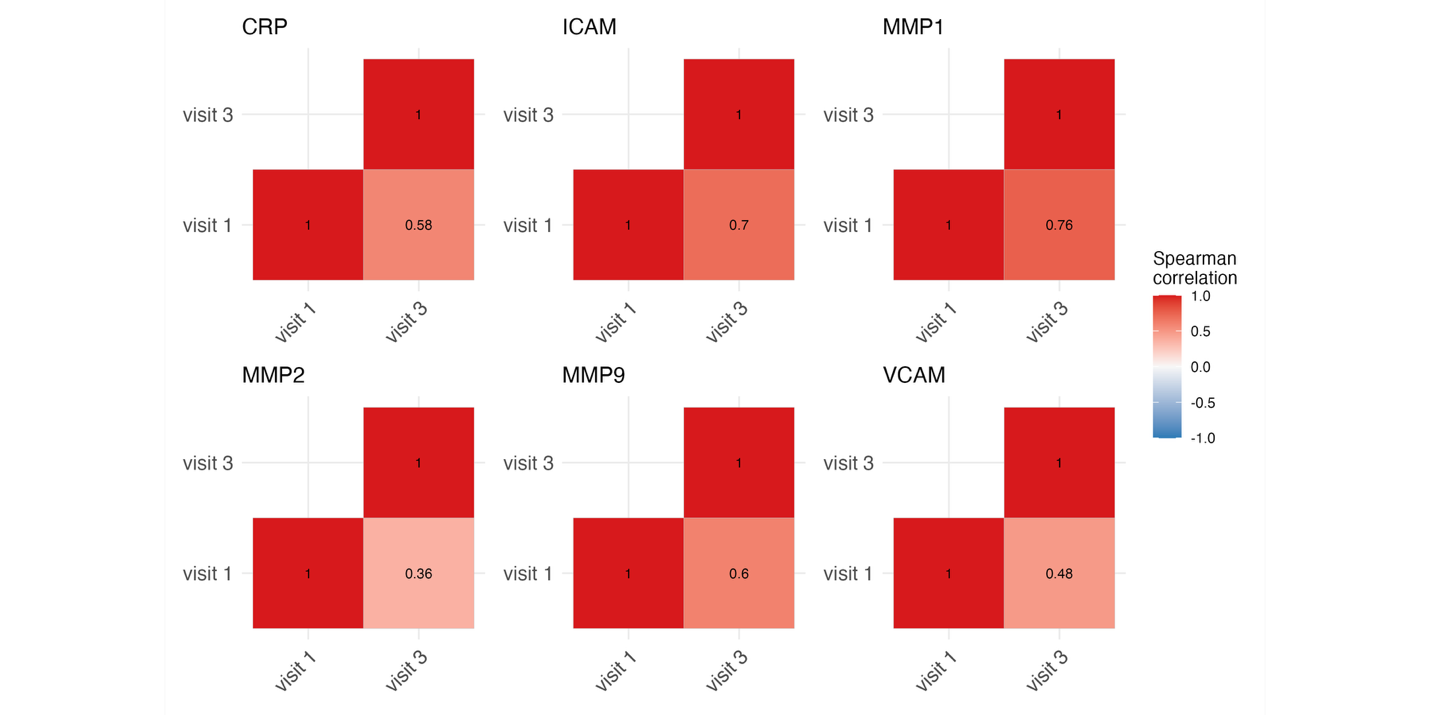


b.


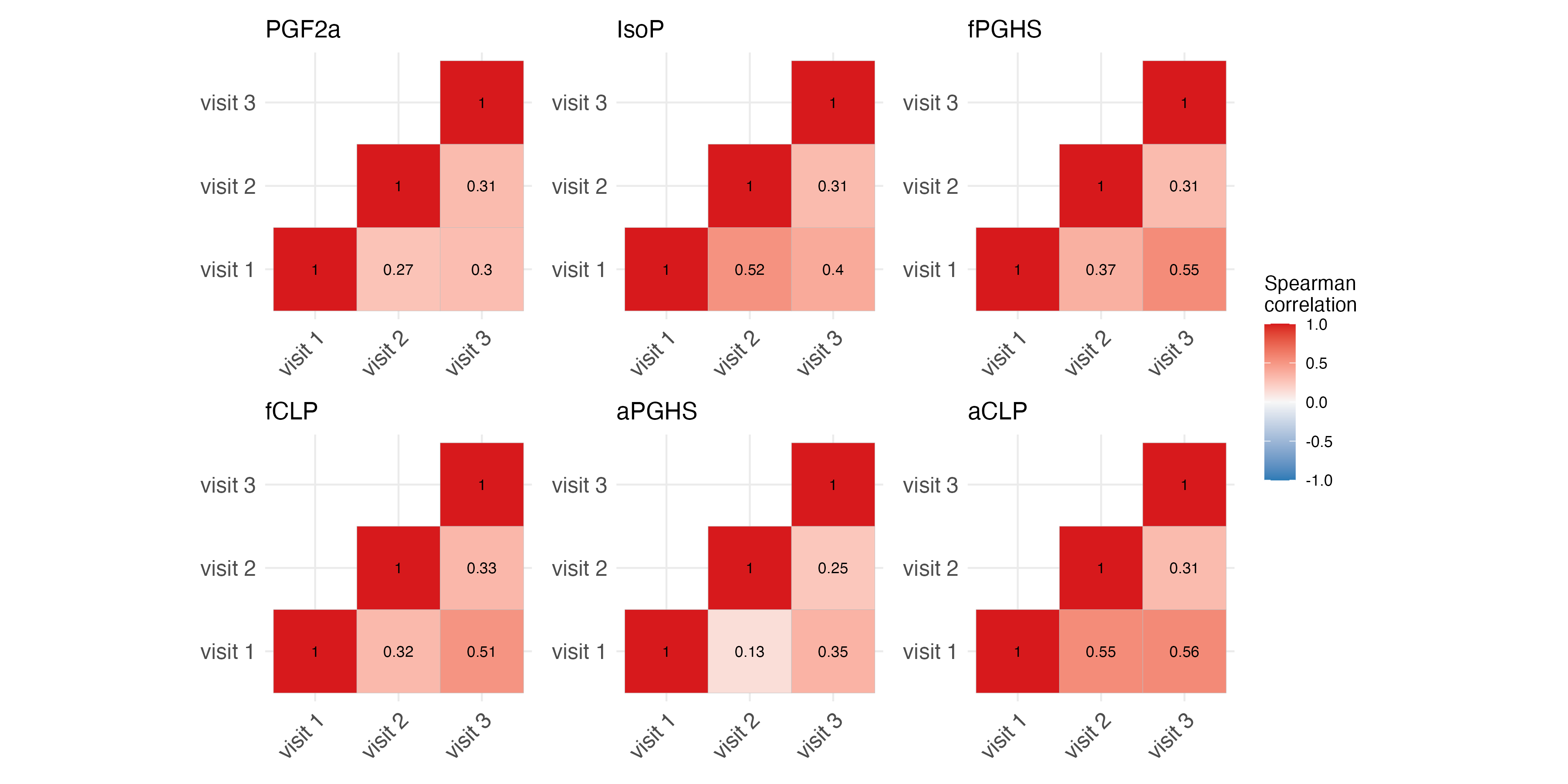


**Supplementary Figure S2.** Spearman correlations among BDI-2 domains are shown as follows: (a) correlations across domains within each child age group in the inflammation analysis sample; (b) correlations across domains within each child age group in the oxidative stress analysis sample. Correlations across domains were not estimated for the 3-year age group because only the Motor domain was assessed. Spearman correlations for the same domain score across age groups are shown for (c) the inflammation analysis sample and (d) the oxidative stress analysis sample.


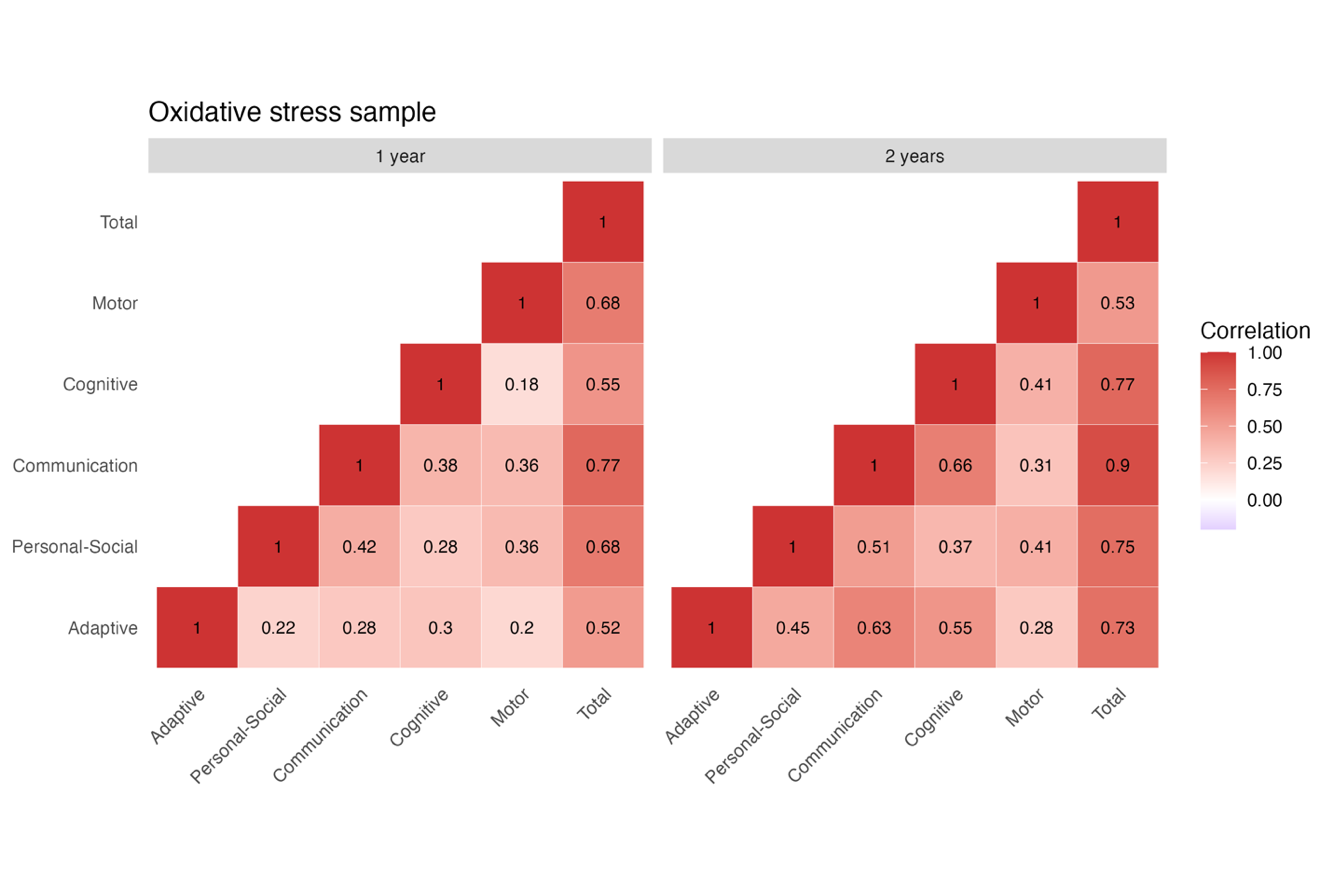


a.


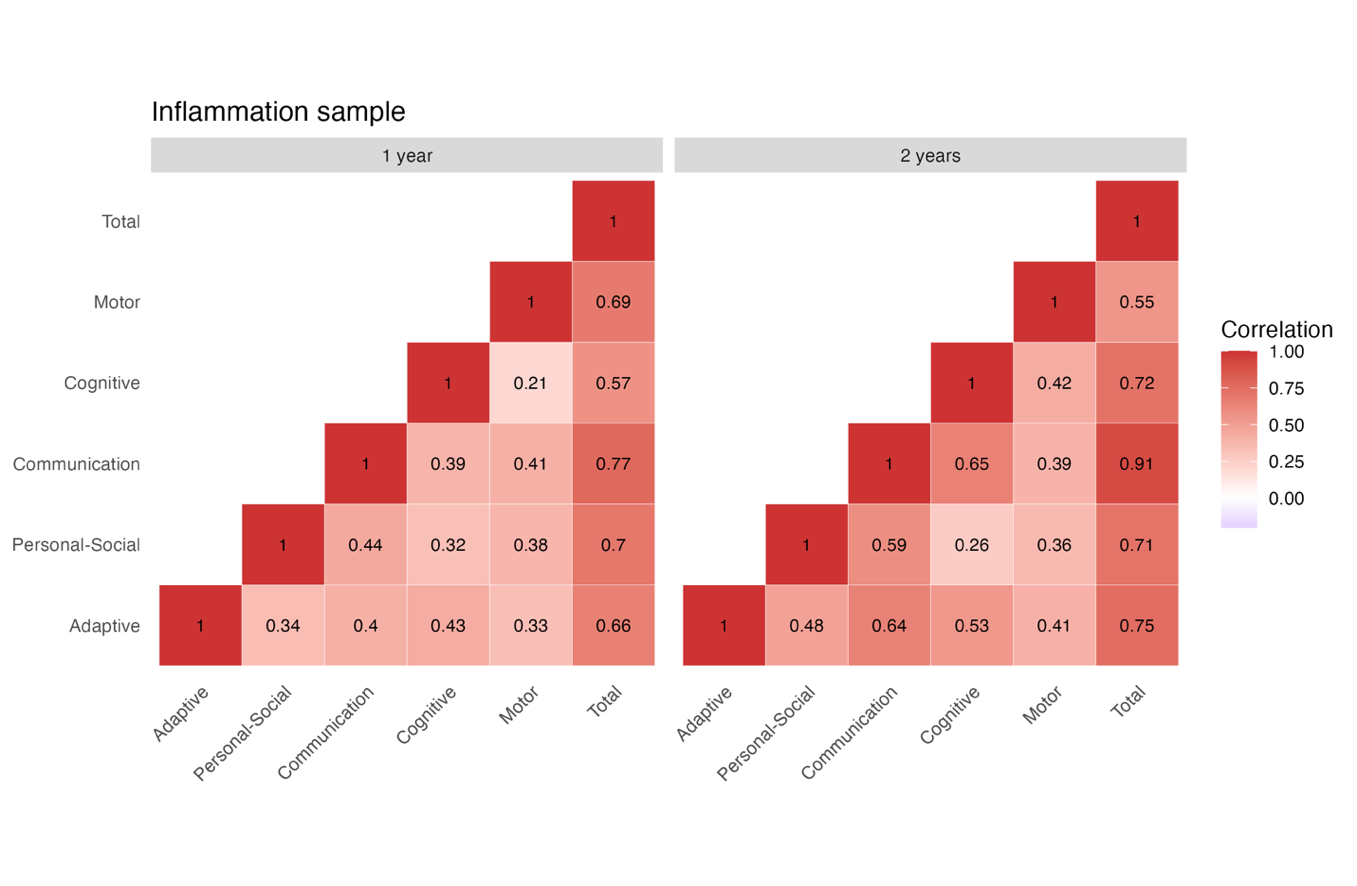


b.


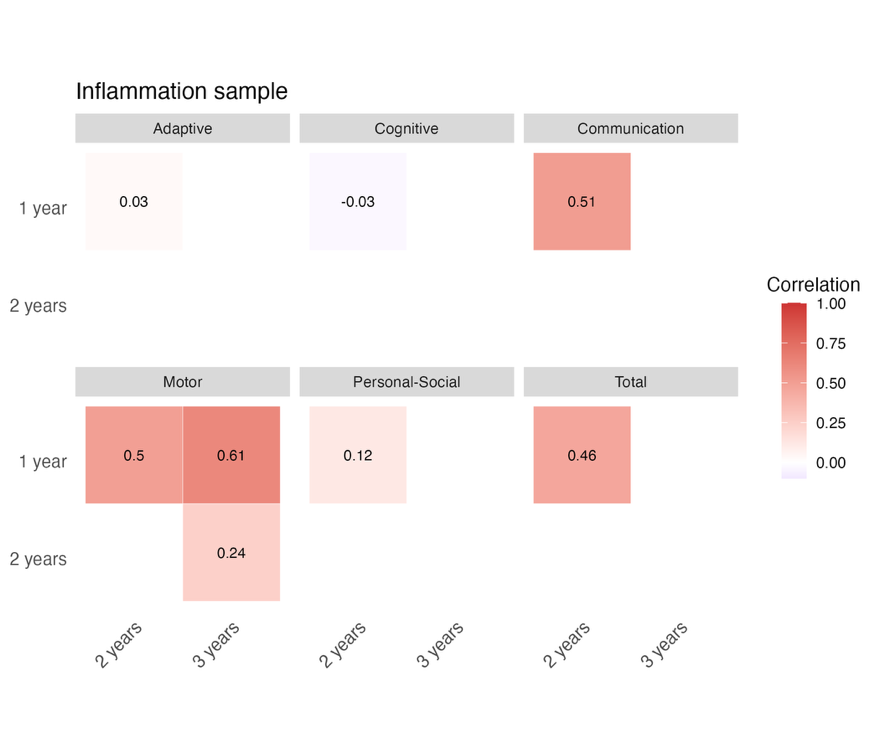


c.

d.


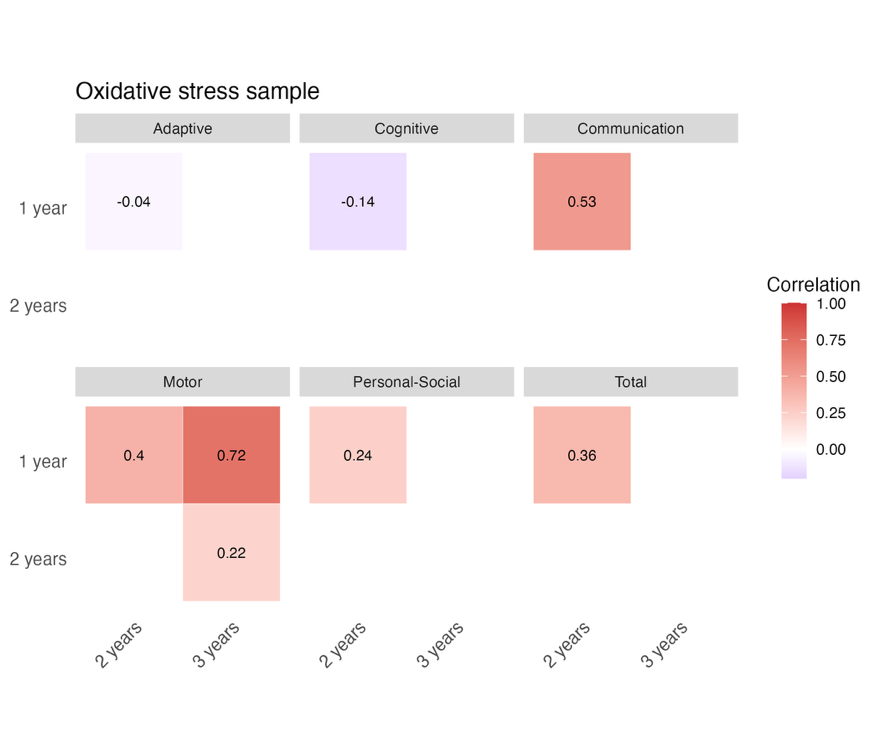


**Supplementary Figure S3.** Percent change in BDI-2 DQ scores for a doubling in gestational geometric mean of inflammation biomarker concentrations by child age group. BDI-2 domain scores were measured at approximately 1 and 2 years of age, with an additional Motor-domain assessment at 3 years. Overall estimates were obtained from linear mixed-effects models with the geometric mean of the maternal biomarker as the predictor and repeated BDI-2 scores as the outcome, adjusted for maternal age, maternal education, pre-pregnancy BMI, child age group, standardized child age in months within each age group, and child sex. Age-group-specific estimates were derived from age-group–stratified linear regression models with gestational geometric mean of biomarker concentrations as the predictor and a single (non-repeated) BDI-2 score as the outcome. In stratified models, the same covariates were included except the child age group. The numbers of participants and observations by each age group are following N=193 (266), N_1year_ =104 (104), N_2 years_=81 (81), N_3 years_=81 (81).


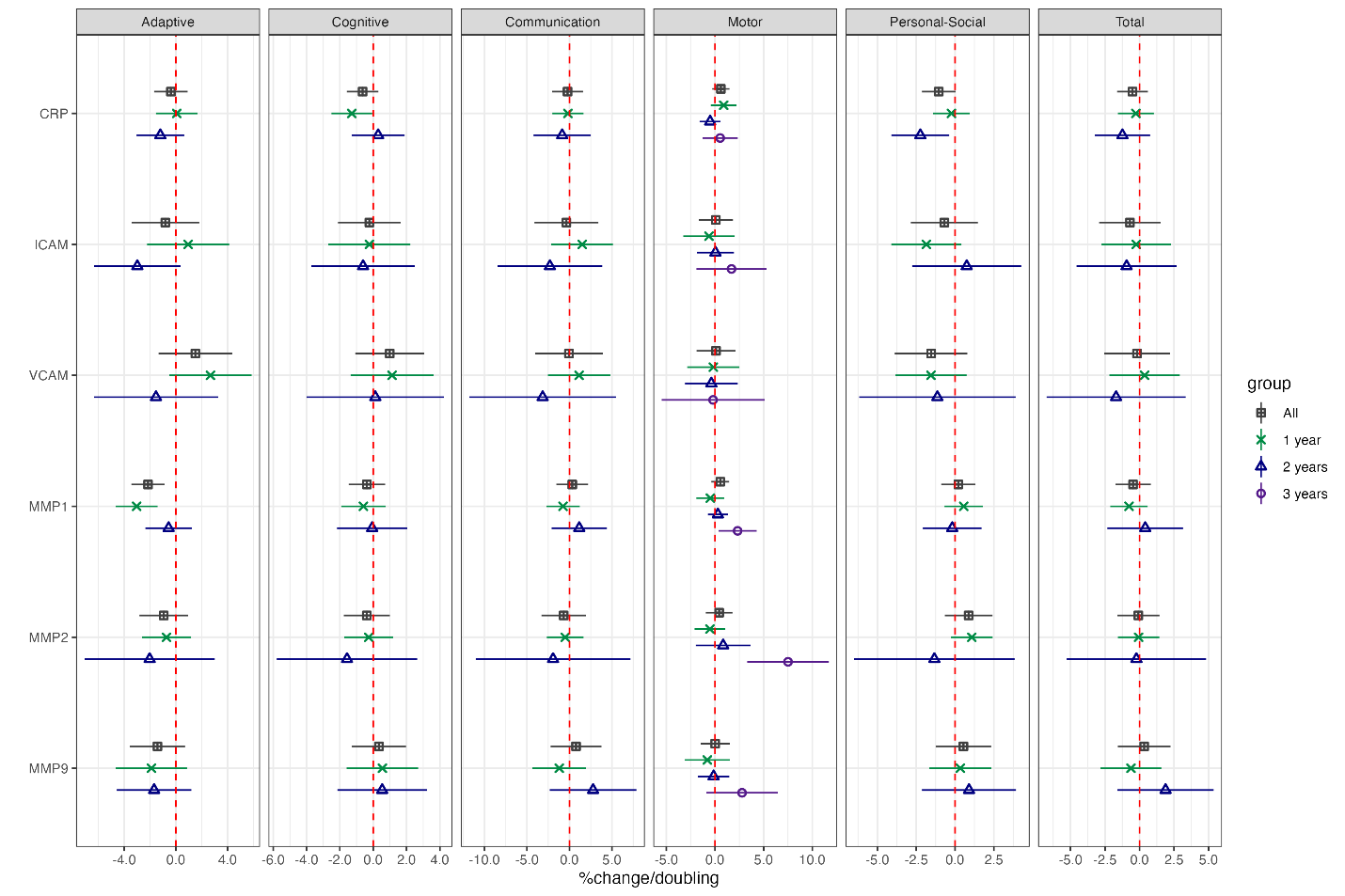


**Supplementary Figure S4.** Percent change in BDI-2 DQ scores for a doubling in gestational geometric mean of oxidative stress biomarker by child age group. BDI-2 domain scores were measured at approximately 1 and 2 years of age, with an additional Motor-domain assessment at 3 years. Overall estimates were obtained from linear mixed-effects models with the geometric mean of the maternal biomarker as the predictor and repeated BDI-2 scores as the outcome, adjusted for maternal age, maternal education, pre-pregnancy BMI, child age group, standardized child age in months within each age group, and child sex. Age-group-specific estimates were derived from age-group–stratified linear regression models with gestational geometric mean of biomarker concentrations as the predictor and a single (non-repeated) BDI-2 score as the outcome. In stratified models, the same covariates were included except the child age group. The numbers of participants and observations by child age are following N_all_=247 (321), N_1year_ =123 (123) N_2 years_=114 (114), N_3 years_=84 (84).


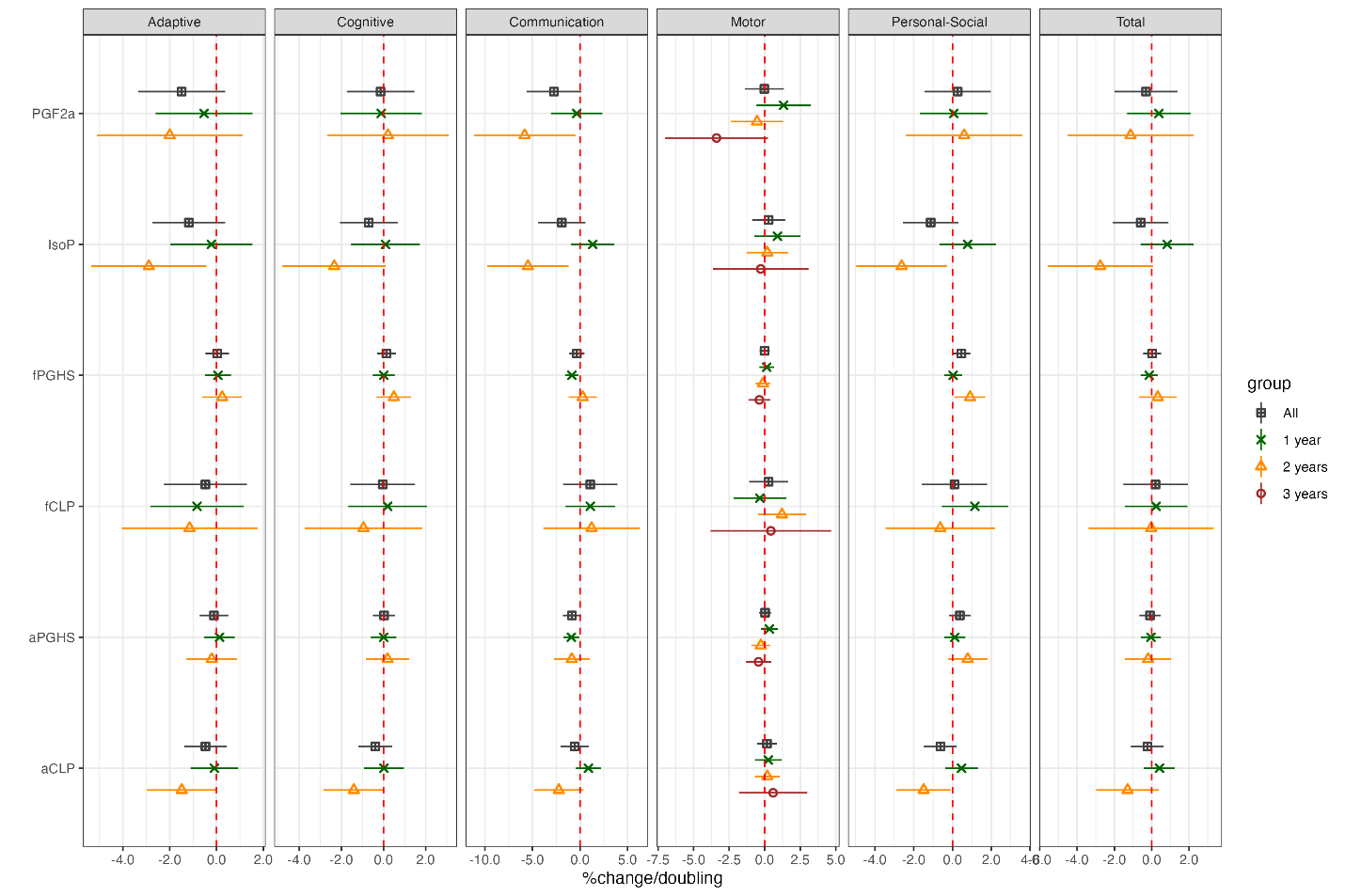


**Supplementary Table S5**. Percent change in BDI-2 DQ scores for a doubling in gestational geometric mean of inflammation biomarker concentration by child sex. BDI-2 domain scores were measured at approximately 1 and 2 years of age, with an additional Motor-domain assessment at 3 years. Linear mixed-effects models with random intercepts were used to account for repeated outcome measurements. Models were adjusted for maternal age (continuous), maternal education (categorical: high school, GED, or less; some college; bachelor’s degree or higher), pre-pregnancy BMI (continuous), child age group (categorical), child age in months (continuous; centered and scaled within each age group), and child sex. Sex-specific estimates were derived from stratified models adjusted for the same covariates, except child sex. Bold text indicates significant interaction term biomarker * child sex (p-value < 0.05).

| marker | group | Adaptive | Cognitive | Communication | Motor | Personal-Social | Total |
| --- | --- | --- | --- | --- | --- | --- | --- |
| CRP | Interaction term (marker*sex)  p-value | 0.948 | 0.377 | 0.874 | 0.164 | **0.003** | 0.183 |
|  | All | -0.39 (-1.69, 0.91) | -0.64 (-1.59, 0.31) | -0.23 (-2.04, 1.59) | 0.6 (-0.28, 1.49) | -1.05 (-2.12, 0.02) | -0.51 (-1.63, 0.61) |
|  | Male | -0.36 (-2.51, 1.78) | -0.81 (-2.46, 0.85) | 0.38 (-2.6, 3.36) | -0.11 (-1.5, 1.29) | -2.88 (-4.61, -1.15) | -1.14 (-2.96, 0.69) |
|  | Female | -0.51 (-2.14, 1.12) | -0.61 (-1.71, 0.5) | -0.81 (-3.04, 1.41) | 0.9 (-0.23, 2.04) | 0.04 (-1.24, 1.32) | -0.25 (-1.66, 1.15) |
| ICAM | Interaction term (marker*sex)  p-value | 0.993 | 0.95 | 0.924 | 0.959 | 0.99 | 0.831 |
|  | All | -0.82 (-3.43, 1.79) | -0.23 (-2.12, 1.65) | -0.36 (-4.13, 3.41) | 0.09 (-1.68, 1.86) | -0.69 (-2.85, 1.47) | -0.7 (-2.93, 1.54) |
|  | Male | -1.23 (-4.95, 2.49) | -1.39 (-4.12, 1.35) | -1.74 (-6.97, 3.49) | -0.86 (-3.15, 1.43) | -1.82 (-4.82, 1.17) | -1.55 (-4.57, 1.48) |
|  | Female | -0.93 (-5.51, 3.64) | -0.71 (-4.09, 2.67) | -0.07 (-6.37, 6.24) | 0.32 (-3, 3.63) | -0.81 (-4.34, 2.72) | -0.94 (-5.03, 3.14) |
| VCAM | Interaction term (marker*sex)  p-value | 0.861 | 0.373 | 0.255 | 0.83 | 0.923 | 0.979 |
|  | All | 1.5 (-1.34, 4.35) | 0.99 (-1.08, 3.06) | -0.07 (-4.06, 3.92) | 0.11 (-1.89, 2.11) | -1.54 (-3.88, 0.79) | -0.18 (-2.56, 2.2) |
|  | Male | 1.77 (-2.14, 5.69) | -0.54 (-3.57, 2.49) | 0.79 (-4.66, 6.25) | -0.79 (-3.37, 1.79) | -2.71 (-5.89, 0.47) | -0.64 (-3.86, 2.58) |
|  | Female | 1.94 (-3.36, 7.23) | 2.84 (-0.83, 6.51) | -3.84 (-11.05, 3.37) | 0.15 (-3.45, 3.74) | -1.92 (-6.09, 2.26) | -0.1 (-4.65, 4.46) |
| MMP1 | Interaction term (marker*sex)  p-value | 0.106 | 0.213 | 0.852 | 0.287 | 0.239 | 0.273 |
|  | All | -2.16 (-3.44, -0.87) | -0.38 (-1.47, 0.72) | 0.33 (-1.54, 2.2) | 0.55 (-0.37, 1.46) | 0.21 (-0.9, 1.32) | -0.47 (-1.76, 0.82) |
|  | Male | -3.52 (-5.59, -1.45) | 0.53 (-1.23, 2.3) | 0.79 (-2.3, 3.88) | 1.19 (-0.19, 2.58) | -0.53 (-2.38, 1.32) | 0.36 (-1.55, 2.27) |
|  | Female | -1.01 (-2.74, 0.72) | -1.04 (-2.47, 0.4) | 0.51 (-1.88, 2.9) | 0.27 (-0.97, 1.51) | 0.65 (-0.73, 2.02) | -1.1 (-2.91, 0.72) |
| MMP2 | Interaction term (marker*sex)  p-value | 0.809 | 0.993 | 0.82 | 0.486 | 0.613 | 0.967 |
|  | All | -0.94 (-2.82, 0.93) | -0.38 (-1.76, 1) | -0.69 (-3.3, 1.93) | 0.43 (-0.94, 1.8) | 0.88 (-0.66, 2.41) | -0.09 (-1.62, 1.44) |
|  | Male | -1.22 (-3.87, 1.43) | -0.23 (-2.25, 1.79) | -1.23 (-4.94, 2.48) | 0.91 (-0.88, 2.71) | 0.77 (-1.42, 2.97) | -0.14 (-2.27, 2) |
|  | Female | -0.55 (-3.51, 2.41) | -0.25 (-2.3, 1.81) | 0.54 (-3.46, 4.55) | 0.26 (-1.93, 2.46) | 2.08 (-0.21, 4.36) | 0.44 (-2.01, 2.88) |
| MMP9 | Interaction term (marker*sex)  p-value | 0.608 | 0.515 | 0.259 | 0.785 | 0.195 | 0.926 |
|  | All | -1.43 (-3.56, 0.71) | 0.34 (-1.28, 1.97) | 0.77 (-2.22, 3.76) | 0.02 (-1.48, 1.52) | 0.54 (-1.24, 2.32) | 0.34 (-1.57, 2.24) |
|  | Male | -1.71 (-5.05, 1.64) | -0.06 (-2.64, 2.52) | -1.64 (-6.25, 2.98) | -0.46 (-2.69, 1.76) | -1.15 (-3.92, 1.63) | -0.29 (-3.18, 2.6) |
|  | Female | -2.5 (-5.37, 0.37) | 0.72 (-1.52, 2.96) | 2.06 (-1.95, 6.07) | 0.17 (-1.94, 2.29) | 2.27 (-0.02, 4.56) | 0.41 (-2.33, 3.14) |

**Supplementary Table S6**. Percent change in BDI-2 DQ scores for a doubling in gestational-visit-specific inflammation biomarker concentration. BDI-2 domain scores were measured at approximately 1 and 2 years of age, with an additional Motor-domain assessment at 3 years. Linear mixed-effects models with random intercepts were used to account for repeated outcome measurements. Models were adjusted for maternal age (continuous), maternal education (categorical: high school, GED, or less; some college; bachelor’s degree or higher), pre-pregnancy BMI (continuous), child age group (categorical), child age in months (continuous; centered and scaled within each age group), and child sex. Gestational-visit-specific estimates were derived from stratified models adjusted for the same covariates. Bold text indicates significant interaction term biomarker * gestational study visit (p-value < 0.05).

| marker | group | Adaptive | Cognitive | Communication | Motor | Personal-Social | Total |
| --- | --- | --- | --- | --- | --- | --- | --- |
| CRP | Interaction term (marker*visit 3) p-value | 0.638 | 0.891 | 0.956 | 0.78 | 0.876 | 0.852 |
|  | visit 1 | -0.09 (-1.48, 1.3) | -0.74 (-1.82, 0.34) | -0.25 (-2.24, 1.74) | 0.69 (-0.27, 1.65) | -0.85 (-1.98, 0.28) | -0.43 (-1.68, 0.82) |
|  | visit 3 | -0.64 (-2.31, 1.03) | -0.55 (-1.73, 0.64) | -0.45 (-2.62, 1.72) | 0.45 (-0.57, 1.46) | -0.84 (-2.16, 0.49) | -0.38 (-1.68, 0.91) |
| ICAM | Interaction term (marker*visit 3) p-value | 0.607 | 0.724 | 0.938 | 0.86 | 0.523 | 0.843 |
|  | visit 1 | -1.47 (-4.55, 1.6) | -0.02 (-2.31, 2.26) | -0.47 (-5.09, 4.15) | 0.32 (-1.75, 2.39) | -1.63 (-4.05, 0.79) | -1.06 (-3.78, 1.65) |
|  | visit 3 | 0.28 (-3.55, 4.1) | -0.03 (-2.88, 2.82) | -0.75 (-5.74, 4.24) | 0.86 (-1.37, 3.1) | 0.32 (-2.74, 3.37) | -0.01 (-3.09, 3.07) |
| VCAM | Interaction term (marker*visit 3) p-value | 0.53 | 0.768 | 0.998 | 0.555 | 0.593 | 0.742 |
|  | visit 1 | 1.17 (-1.77, 4.11) | 1.58 (-0.63, 3.8) | 0.56 (-3.68, 4.79) | 0.2 (-1.92, 2.33) | -1.05 (-3.41, 1.31) | 0.08 (-2.44, 2.6) |
|  | visit 3 | 3.07 (-1.61, 7.74) | 0.6 (-2.82, 4.02) | -0.91 (-6.93, 5.11) | 1.06 (-1.76, 3.87) | -0.75 (-4.49, 2.99) | 0.48 (-3.22, 4.19) |
| MMP1 | Interaction term (marker*visit 3) p-value | 0.515 | 0.904 | 0.739 | 0.674 | 0.659 | 0.952 |
|  | visit 1 | -1.54 (-2.8, -0.28) | -0.44 (-1.51, 0.63) | 0.39 (-1.46, 2.24) | 0.56 (-0.35, 1.46) | 0.31 (-0.73, 1.34) | -0.26 (-1.5, 0.98) |
|  | visit 3 | -3.39 (-5.44, -1.35) | -0.95 (-2.66, 0.76) | -1.49 (-4.28, 1.3) | 0.78 (-0.54, 2.1) | -0.66 (-2.42, 1.1) | -1.07 (-2.98, 0.84) |
| MMP2 | Interaction term (marker*visit 3) p-value | 0.99 | 0.68 | 0.935 | 0.627 | 0.87 | 0.986 |
|  | visit 1 | -0.92 (-2.85, 1) | -0.58 (-2.06, 0.89) | -0.85 (-3.58, 1.89) | 0.27 (-1.15, 1.68) | 0.64 (-0.91, 2.19) | -0.17 (-1.77, 1.43) |
|  | visit 3 | -1.05 (-4.93, 2.82) | 0.33 (-2.52, 3.17) | -0.6 (-5.59, 4.39) | -0.66 (-3.25, 1.94) | 1.3 (-1.8, 4.39) | 0 (-3.01, 3.01) |
| MMP9 | Interaction term (marker*visit 3) p-value | 0.612 | 0.973 | 0.578 | 0.9 | 0.445 | 0.982 |
|  | visit 1 | -0.53 (-2.76, 1.7) | 0.27 (-1.51, 2.04) | 1.29 (-1.9, 4.49) | -0.15 (-1.76, 1.47) | 1.3 (-0.47, 3.08) | 0.55 (-1.48, 2.58) |
|  | visit 3 | -2.44 (-5.35, 0.48) | 0.93 (-1.34, 3.2) | -1.15 (-4.93, 2.63) | 0.67 (-1.17, 2.51) | -0.58 (-2.94, 1.79) | 1.05 (-1.65, 3.76) |

**Supplementary Table S7**. Percent change in BDI-2 DQ scores for a doubling in gestational geometric mean of inflammation biomarker concentration by child age groups. BDI-2 domain scores were measured at approximately 1 and 2 years of age, with an additional Motor-domain assessment at 3 years. Overall estimates were obtained from linear mixed-effects models with the geometric mean of the maternal biomarker as the predictor and repeated BDI-2 scores as the outcome, adjusted for maternal age, maternal education, pre-pregnancy BMI, child age group, standardized child age in months within each age group, and child sex. Age-group-specific estimates were derived from age-group–stratified linear regression models with gestational geometric mean of biomarker concentrations as the predictor and a single (non-repeated) BDI-2 score as the outcome. In stratified models, the same covariates were included except the child age group. Bold text indicates significant interaction term biomarker * child age group (p-value < 0.05).

| marker | group | Adaptive | Cognitive | Communication | Motor | Personal-Social | Total |
| --- | --- | --- | --- | --- | --- | --- | --- |
| CRP | Interaction term (marker*2 years) p-value | 0.13 | 0.132 | 0.347 | 0.35 | 0.192 | 0.452 |
|  | Interaction term (marker*3 years) p-value |  |  |  | 0.775 |  |  |
|  | All | -0.39 (-1.69, 0.91) | -0.64 (-1.59, 0.31) | -0.23 (-2.04, 1.59) | 0.6 (-0.28, 1.49) | -1.05 (-2.12, 0.02) | -0.51 (-1.63, 0.61) |
|  | 1 year | 0.06 (-1.55, 1.68) | -1.29 (-2.5, -0.07) | -0.18 (-2.02, 1.65) | 0.89 (-0.43, 2.22) | -0.23 (-1.42, 0.95) | -0.27 (-1.57, 1.04) |
|  | 2 years | -1.2 (-3.05, 0.64) | 0.3 (-1.29, 1.89) | -0.86 (-4.22, 2.5) | -0.5 (-1.58, 0.59) | -2.24 (-4.1, -0.37) | -1.24 (-3.23, 0.76) |
|  | 3 years |  |  |  | 0.53 (-1.28, 2.34) |  |  |
| ICAM | Interaction term (marker*2 years) p-value | 0.054 | 0.708 | 0.185 | 0.989 | 0.223 | 0.489 |
|  | Interaction term (marker*3 years) p-value |  |  |  | 0.827 |  |  |
|  | All | -0.82 (-3.43, 1.79) | -0.23 (-2.12, 1.65) | -0.36 (-4.13, 3.41) | 0.09 (-1.68, 1.86) | -0.69 (-2.85, 1.47) | -0.7 (-2.93, 1.54) |
|  | 1 year | 0.95 (-2.23, 4.12) | -0.23 (-2.69, 2.23) | 1.5 (-2.12, 5.11) | -0.62 (-3.26, 2.02) | -1.85 (-4.11, 0.41) | -0.24 (-2.76, 2.28) |
|  | 2 years | -2.98 (-6.33, 0.36) | -0.61 (-3.71, 2.49) | -2.3 (-8.47, 3.87) | 0.05 (-1.86, 1.95) | 0.75 (-2.76, 4.26) | -0.94 (-4.56, 2.69) |
|  | 3 years |  |  |  | 1.7 (-1.91, 5.31) |  |  |
| VCAM | Interaction term (marker*2 years) p-value | 0.121 | 0.586 | 0.117 | 0.818 | 0.964 | 0.229 |
|  | Interaction term (marker*3 years) p-value |  |  |  | 0.634 |  |  |
|  | All | 1.5 (-1.34, 4.35) | 0.99 (-1.08, 3.06) | -0.07 (-4.06, 3.92) | 0.11 (-1.89, 2.11) | -1.54 (-3.88, 0.79) | -0.18 (-2.56, 2.2) |
|  | 1 year | 2.67 (-0.51, 5.86) | 1.13 (-1.35, 3.61) | 1.13 (-2.54, 4.81) | -0.17 (-2.85, 2.51) | -1.55 (-3.85, 0.74) | 0.37 (-2.18, 2.92) |
|  | 2 years | -1.54 (-6.35, 3.27) | 0.13 (-4, 4.25) | -3.16 (-11.79, 5.48) | -0.37 (-3.09, 2.34) | -1.14 (-6.18, 3.9) | -1.7 (-6.74, 3.34) |
|  | 3 years |  |  |  | -0.2 (-5.48, 5.09) |  |  |
| MMP1 | Interaction term (marker*2 years) p-value | **0.035** | 0.45 | 0.101 | 0.288 | 0.702 | 0.172 |
|  | Interaction term (marker*3 years) p-value |  |  |  | **0.002** |  |  |
|  | All | -2.16 (-3.44, -0.87) | -0.38 (-1.47, 0.72) | 0.33 (-1.54, 2.2) | 0.55 (-0.37, 1.46) | 0.21 (-0.9, 1.32) | -0.47 (-1.76, 0.82) |
|  | 1 year | -3.04 (-4.66, -1.42) | -0.59 (-1.92, 0.74) | -0.76 (-2.72, 1.21) | -0.48 (-1.91, 0.96) | 0.56 (-0.68, 1.8) | -0.76 (-2.12, 0.6) |
|  | 2 years | -0.56 (-2.36, 1.23) | -0.06 (-2.17, 2.04) | 1.15 (-2.1, 4.4) | 0.3 (-0.73, 1.33) | -0.18 (-2.07, 1.71) | 0.41 (-2.33, 3.15) |
|  | 3 years |  |  |  | 2.33 (0.37, 4.29) |  |  |
| MMP2 | Interaction term (marker*2 years) p-value | 0.912 | 0.509 | 0.531 | 0.603 | 0.402 | 0.992 |
|  | Interaction term (marker*3 years) p-value |  |  |  | **0.001** |  |  |
|  | All | -0.94 (-2.82, 0.93) | -0.38 (-1.76, 1) | -0.69 (-3.3, 1.93) | 0.43 (-0.94, 1.8) | 0.88 (-0.66, 2.41) | -0.09 (-1.62, 1.44) |
|  | 1 year | -0.73 (-2.62, 1.16) | -0.27 (-1.74, 1.2) | -0.51 (-2.67, 1.66) | -0.52 (-2.1, 1.05) | 1.08 (-0.27, 2.42) | -0.07 (-1.57, 1.43) |
|  | 2 years | -2.03 (-7.07, 3.01) | -1.57 (-5.78, 2.64) | -1.93 (-11.04, 7.18) | 0.84 (-1.97, 3.65) | -1.33 (-6.5, 3.84) | -0.23 (-5.28, 4.82) |
|  | 3 years |  |  |  | 7.51 (3.31, 11.7) |  |  |
| MMP9 | Interaction term (marker*2 years) p-value | 0.928 | 0.643 | 0.32 | 0.914 | 0.422 | 0.316 |
|  | Interaction term (marker*3 years) p-value |  |  |  | 0.131 |  |  |
|  | All | -1.43 (-3.56, 0.71) | 0.34 (-1.28, 1.97) | 0.77 (-2.22, 3.76) | 0.02 (-1.48, 1.52) | 0.54 (-1.24, 2.32) | 0.34 (-1.57, 2.24) |
|  | 1 year | -1.89 (-4.65, 0.87) | 0.55 (-1.6, 2.7) | -1.2 (-4.37, 1.96) | -0.79 (-3.1, 1.52) | 0.34 (-1.66, 2.34) | -0.62 (-2.82, 1.58) |
|  | 2 years | -1.68 (-4.57, 1.2) | 0.54 (-2.15, 3.23) | 2.78 (-2.32, 7.88) | -0.14 (-1.76, 1.48) | 0.9 (-2.13, 3.92) | 1.88 (-1.6, 5.37) |
|  | 3 years |  |  |  | 2.78 (-0.9, 6.46) |  |  |

**Supplementary Table S8**. Percent change in BDI-2 DQ scores for a doubling in gestational geometric mean of oxidative stress biomarker concentration by child sex. BDI-2 domain scores were measured at approximately 1 and 2 years of age, with an additional Motor-domain assessment at 3 years. Linear mixed-effects models with random intercepts were used to account for repeated outcome measurements. Models were adjusted for maternal age (continuous), maternal education (categorical: high school, GED, or less; some college; bachelor’s degree or higher), pre-pregnancy BMI (continuous), child age group (categorical), child age in months (continuous; centered and scaled within each age group), and child sex. Sex-specific estimates were derived from stratified models adjusted for the same covariates, except child sex. Bold text indicates significant interaction term biomarker * child sex (p-value < 0.05).

| marker | sex | Adaptive | Cognitive | Communication | Motor | Personal-Social | Total |
| --- | --- | --- | --- | --- | --- | --- | --- |
| PGF2a | Interaction term (marker*sex)  p-value | 0.55 | 0.97 | 0.772 | 0.486 | 0.965 | 0.864 |
|  | All | -1.49 (-3.35, 0.37) | -0.14 (-1.75, 1.47) | -2.74 (-5.63, 0.15) | -0.03 (-1.41, 1.35) | 0.27 (-1.44, 1.98) | -0.31 (-1.99, 1.38) |
|  | Male | -1.08 (-4, 1.85) | -0.16 (-2.74, 2.42) | -1.33 (-5.78, 3.12) | -0.64 (-2.63, 1.36) | 0.1 (-2.53, 2.72) | 0.03 (-2.46, 2.52) |
|  | Female | -1.4 (-3.79, 1) | -0.19 (-2.24, 1.86) | -3.75 (-7.57, 0.07) | 0.75 (-1.19, 2.69) | 0.25 (-2.05, 2.55) | -0.45 (-2.86, 1.96) |
| IsoP | Interaction term (marker*sex)  p-value | **0.021** | 0.193 | 0.077 | 0.268 | **0.012** | **0.039** |
|  | All | -1.18 (-2.73, 0.37) | -0.7 (-2.07, 0.67) | -1.92 (-4.41, 0.58) | 0.28 (-0.89, 1.44) | -1.13 (-2.56, 0.31) | -0.59 (-2.08, 0.9) |
|  | Male | -2.96 (-5.43, -0.49) | -1.5 (-3.71, 0.72) | -3.68 (-7.55, 0.2) | -0.43 (-2.17, 1.32) | -3.21 (-5.35, -1.07) | -1.97 (-4.21, 0.27) |
|  | Female | 0.28 (-1.8, 2.36) | -0.2 (-2.05, 1.65) | -0.59 (-4.06, 2.89) | 0.89 (-0.77, 2.54) | 0.81 (-1.19, 2.82) | 0.63 (-1.58, 2.84) |
| fPGHS | Interaction term (marker*sex)  p-value | 0.057 | 0.221 | 0.132 | 0.375 | **0.021** | **0.041** |
|  | All | 0.03 (-0.48, 0.54) | 0.14 (-0.31, 0.58) | -0.33 (-1.14, 0.47) | 0 (-0.36, 0.36) | 0.46 (-0.01, 0.92) | 0.02 (-0.46, 0.5) |
|  | Male | 0.89 (0, 1.78) | 0.5 (-0.27, 1.27) | 0.57 (-0.84, 1.97) | 0.28 (-0.33, 0.88) | 1.16 (0.38, 1.94) | 0.73 (-0.04, 1.51) |
|  | Female | -0.42 (-1.01, 0.17) | -0.05 (-0.58, 0.47) | -0.77 (-1.73, 0.19) | -0.1 (-0.56, 0.35) | -0.01 (-0.58, 0.57) | -0.41 (-1.04, 0.23) |
| fCLP | Interaction term (marker*sex)  p-value | 0.336 | 0.595 | 0.248 | 0.994 | 0.269 | 0.403 |
|  | All | -0.47 (-2.25, 1.3) | -0.04 (-1.58, 1.5) | 1.07 (-1.79, 3.94) | 0.27 (-1.09, 1.64) | 0.1 (-1.58, 1.79) | 0.2 (-1.52, 1.93) |
|  | Male | -2.34 (-5.35, 0.67) | -0.54 (-3.23, 2.14) | -1.37 (-6.07, 3.33) | 0.41 (-1.71, 2.53) | -0.9 (-3.6, 1.8) | -0.92 (-3.73, 1.89) |
|  | Female | 0.39 (-1.74, 2.52) | 0.2 (-1.6, 2.01) | 2.65 (-0.9, 6.19) | -0.12 (-1.93, 1.7) | 0.86 (-1.3, 3.02) | 0.82 (-1.43, 3.08) |
| aPGHS | Interaction term (marker*sex)  p-value | 0.263 | 0.464 | 0.367 | 0.576 | 0.206 | 0.168 |
|  | All | -0.11 (-0.72, 0.5) | 0.03 (-0.5, 0.56) | -0.84 (-1.8, 0.12) | 0.03 (-0.4, 0.46) | 0.38 (-0.18, 0.94) | -0.09 (-0.66, 0.48) |
|  | Male | 0.78 (-0.33, 1.88) | 0.36 (-0.59, 1.3) | 0.06 (-1.65, 1.76) | 0.29 (-0.44, 1.03) | 0.83 (-0.15, 1.81) | 0.62 (-0.32, 1.56) |
|  | Female | -0.5 (-1.19, 0.19) | -0.13 (-0.74, 0.48) | -1.22 (-2.34, -0.09) | -0.03 (-0.57, 0.51) | 0.09 (-0.58, 0.77) | -0.47 (-1.2, 0.26) |
| aCLP | Interaction term (marker*sex)  p-value | **0.034** | 0.207 | 0.073 | 0.387 | **0.007** | **0.048** |
|  | All | -0.47 (-1.39, 0.44) | -0.39 (-1.18, 0.4) | -0.56 (-2.04, 0.93) | 0.16 (-0.55, 0.87) | -0.63 (-1.48, 0.23) | -0.23 (-1.11, 0.65) |
|  | Male | -1.65 (-3.15, -0.15) | -0.86 (-2.15, 0.43) | -1.88 (-4.23, 0.48) | -0.12 (-1.19, 0.94) | -1.91 (-3.2, -0.62) | -1.15 (-2.52, 0.22) |
|  | Female | 0.37 (-0.83, 1.57) | -0.1 (-1.15, 0.94) | 0.38 (-1.64, 2.4) | 0.37 (-0.64, 1.37) | 0.55 (-0.61, 1.72) | 0.47 (-0.8, 1.73) |

**Supplementary Table S9.** Percent change in BDI-2 DQ scores for a doubling in gestational-visit-specific oxidative stress biomarker concentration. BDI-2 domain scores were measured at approximately 1 and 2 years of age, with an additional Motor-domain assessment at 3 years. Linear mixed-effects models with random intercepts were used to account for repeated outcome measurements. Models were adjusted for maternal age (continuous), maternal education (categorical: high school, GED, or less; some college; bachelor’s degree or higher), pre-pregnancy BMI (continuous), child age group (categorical), child age in months (continuous; centered and scaled within each age group), and child sex. Gestational-visit-specific estimates were derived from stratified models adjusted for the same covariates. Bold text indicates significant interaction term biomarker * gestational visit (p-value < 0.05).

| marker | group | Adaptive | Cognitive | Communication | Motor | Personal-Social | Total |
| --- | --- | --- | --- | --- | --- | --- | --- |
| PGF2a | Interaction term (marker*visit 2)  p-value | 0.809 | 0.896 | 0.972 | 0.737 | 0.887 | 0.953 |
|  | Interaction term (marker*visit 3)  p-value | 0.791 | 0.874 | 0.978 | 0.78 | 0.814 | 0.907 |
|  | visit 1 | -0.74 (-2.2, 0.72) | -0.74 (-1.91, 0.42) | -1.28 (-3.33, 0.78) | 0.59 (-0.39, 1.57) | 0.39 (-0.89, 1.67) | -0.41 (-1.63, 0.81) |
|  | visit 2 | 0.46 (-1.2, 2.11) | 0.21 (-1.19, 1.6) | -1.84 (-4.39, 0.7) | -0.5 (-1.65, 0.66) | 0.1 (-1.4, 1.6) | 0.11 (-1.39, 1.6) |
|  | visit 3 | -2.42 (-4.16, -0.69) | -0.67 (-2.21, 0.88) | -2.7 (-5.58, 0.18) | -0.67 (-2.06, 0.72) | -1.08 (-2.75, 0.59) | -0.9 (-2.56, 0.76) |
| IsoP | Interaction term (marker*visit 2)  p-value | 0.71 | 0.875 | 0.801 | 0.999 | 0.771 | 0.911 |
|  | Interaction term (marker*visit 3)  p-value | 0.934 | 0.986 | 0.981 | 0.846 | 0.978 | 0.873 |
|  | visit 1 | -1.14 (-2.85, 0.56) | -0.26 (-1.65, 1.12) | 0.16 (-2.27, 2.59) | 0.1 (-1.05, 1.25) | -0.17 (-1.7, 1.35) | -0.21 (-1.67, 1.25) |
|  | visit 2 | 0.09 (-1.27, 1.46) | -0.78 (-1.93, 0.36) | -2.34 (-4.49, -0.18) | -0.29 (-1.28, 0.69) | -1.48 (-2.72, -0.24) | -0.82 (-2.08, 0.44) |
|  | visit 3 | -1.09 (-2.55, 0.37) | -0.27 (-1.64, 1.09) | -0.6 (-2.96, 1.76) | 0.51 (-0.62, 1.65) | -0.66 (-2.01, 0.69) | 0.04 (-1.4, 1.49) |
| fPGHS | Interaction term (marker*visit 2)  p-value | 0.974 | 0.676 | 0.781 | 0.766 | 0.838 | 0.727 |
|  | Interaction term (marker*visit 3)  p-value | 0.829 | 0.96 | 0.959 | 0.68 | 0.85 | 0.873 |
|  | visit 1 | 0.1 (-0.39, 0.59) | -0.16 (-0.55, 0.22) | -0.44 (-1.13, 0.25) | 0.2 (-0.12, 0.52) | 0.32 (-0.11, 0.75) | -0.13 (-0.54, 0.29) |
|  | visit 2 | 0.07 (-0.36, 0.51) | 0.3 (-0.07, 0.66) | 0.18 (-0.51, 0.87) | 0.06 (-0.26, 0.37) | 0.55 (0.16, 0.94) | 0.31 (-0.09, 0.7) |
|  | visit 3 | -0.32 (-0.79, 0.15) | -0.15 (-0.6, 0.29) | -0.48 (-1.26, 0.3) | -0.17 (-0.53, 0.18) | -0.06 (-0.51, 0.39) | -0.28 (-0.75, 0.18) |
| fCLP | Interaction term (marker*visit 2)  p-value | 0.899 | 0.803 | 0.874 | 0.764 | 0.92 | 0.935 |
|  | Interaction term (marker*visit 3)  p-value | 0.846 | 0.873 | 0.993 | 0.568 | 0.827 | 0.905 |
|  | visit 1 | -0.9 (-2.89, 1.08) | 0.91 (-0.71, 2.53) | 1.83 (-0.98, 4.63) | -0.82 (-2.17, 0.53) | -0.39 (-2.18, 1.39) | 0.23 (-1.52, 1.98) |
|  | visit 2 | -0.29 (-1.82, 1.25) | -0.19 (-1.5, 1.12) | -0.32 (-2.75, 2.1) | -0.05 (-1.18, 1.08) | -0.09 (-1.52, 1.34) | -0.37 (-1.82, 1.08) |
|  | visit 3 | 0.41 (-1.15, 1.96) | 0.19 (-1.17, 1.55) | 2.59 (0.08, 5.09) | 1.12 (-0.09, 2.33) | 0.97 (-0.5, 2.43) | 0.94 (-0.52, 2.39) |
| aPGHS | Interaction term (marker*visit 2)  p-value | 0.958 | 0.724 | 0.863 | 0.739 | 0.955 | 0.758 |
|  | Interaction term (marker*visit 3)  p-value | 0.785 | 0.912 | 0.972 | 0.642 | 0.766 | 0.85 |
|  | visit 1 | 0.07 (-0.44, 0.57) | -0.23 (-0.63, 0.17) | -0.43 (-1.15, 0.29) | 0.25 (-0.08, 0.59) | 0.33 (-0.12, 0.77) | -0.14 (-0.57, 0.29) |
|  | visit 2 | 0.11 (-0.36, 0.58) | 0.22 (-0.18, 0.62) | -0.08 (-0.83, 0.67) | 0.04 (-0.3, 0.38) | 0.4 (-0.03, 0.83) | 0.25 (-0.18, 0.68) |
|  | visit 3 | -0.57 (-1.1, -0.05) | -0.26 (-0.75, 0.23) | -0.95 (-1.83, -0.07) | -0.22 (-0.62, 0.19) | -0.3 (-0.81, 0.21) | -0.43 (-0.94, 0.09) |
| aCLP | Interaction term (marker*visit 2)  p-value | 0.758 | 0.822 | 0.787 | 0.879 | 0.893 | 0.91 |
|  | Interaction term (marker*visit 3)  p-value | 0.863 | 0.951 | 0.945 | 0.759 | 0.944 | 0.902 |
|  | visit 1 | -0.61 (-1.86, 0.64) | 0.03 (-0.99, 1.05) | 0.97 (-0.8, 2.74) | -0.14 (-0.98, 0.69) | -0.24 (-1.36, 0.88) | 0.05 (-1.03, 1.13) |
|  | visit 2 | 0 (-0.81, 0.81) | -0.44 (-1.12, 0.24) | -0.99 (-2.28, 0.31) | -0.1 (-0.71, 0.51) | -0.79 (-1.53, -0.05) | -0.42 (-1.18, 0.33) |
|  | visit 3 | -0.36 (-1.26, 0.54) | -0.14 (-0.97, 0.69) | -0.02 (-1.46, 1.43) | 0.41 (-0.3, 1.11) | -0.28 (-1.1, 0.54) | 0.13 (-0.74, 1) |

**Supplementary Table S10.** Percent change in BDI-2 DQ scores for a doubling in gestational geometric mean of oxidative stress by child age groups. BDI-2 domain scores were measured at approximately 1 and 2 years of age, with an additional Motor-domain assessment at 3 years. Overall estimates were obtained from linear mixed-effects models with the geometric mean of the maternal biomarker as the predictor and repeated BDI-2 scores as the outcome, adjusted for maternal age, maternal education, pre-pregnancy BMI, child age group, standardized child age in months within each age group, and child sex. Age-group-specific estimates were derived from age-group–stratified linear regression models with gestational geometric mean of biomarker concentrations as the predictor and a single (non-repeated) BDI-2 score as the outcome. In stratified models, the same covariates were included except the child age group. Bold text indicates significant interaction term biomarker * child age group (p-value < 0.05).

| marker | group | Adaptive | Cognitive | Communication | Motor | Personal-Social | Total |
| --- | --- | --- | --- | --- | --- | --- | --- |
| PGF2a | Interaction term (marker*2 years), p-value | 0.256 | 0.778 | 0.076 | 0.108 | 0.645 | 0.196 |
|  | Interaction term (marker*3 years), p-value |  |  |  | **0.001** |  |  |
|  | All | -1.49 (-3.35, 0.37) | -0.14 (-1.75, 1.47) | -2.74 (-5.63, 0.15) | -0.03 (-1.41, 1.35) | 0.27 (-1.44, 1.98) | -0.31 (-1.99, 1.38) |
|  | 1 year | -0.53 (-2.6, 1.54) | -0.11 (-2.04, 1.82) | -0.35 (-3.05, 2.35) | 1.33 (-0.58, 3.24) | 0.06 (-1.68, 1.8) | 0.38 (-1.32, 2.08) |
|  | 2 years | -2 (-5.1, 1.11) | 0.21 (-2.67, 3.09) | -5.82 (-11.17, -0.48) | -0.54 (-2.37, 1.3) | 0.6 (-2.4, 3.61) | -1.14 (-4.51, 2.24) |
|  | 3 years |  |  |  | -3.39 (-7.01, 0.23) |  |  |
| IsoP | Interaction term (marker*2 years), p-value | 0.107 | 0.08 | **0.012** | 0.497 | 0.208 | **0.05** |
|  | Interaction term (marker*3 years), p-value |  |  |  | 0.156 |  |  |
|  | All | -1.18 (-2.73, 0.37) | -0.7 (-2.07, 0.67) | -1.92 (-4.41, 0.58) | 0.28 (-0.89, 1.44) | -1.13 (-2.56, 0.31) | -0.59 (-2.08, 0.9) |
|  | 1 year | -0.22 (-1.98, 1.53) | 0.09 (-1.54, 1.73) | 1.34 (-0.93, 3.62) | 0.9 (-0.72, 2.52) | 0.78 (-0.68, 2.23) | 0.83 (-0.6, 2.25) |
|  | 2 years | -2.89 (-5.35, -0.43) | -2.34 (-4.79, 0.12) | -5.48 (-9.75, -1.21) | 0.18 (-1.28, 1.65) | -2.62 (-4.96, -0.29) | -2.75 (-5.58, 0.07) |
|  | 3 years |  |  |  | -0.27 (-3.64, 3.09) |  |  |
| fPGHS | Interaction term (marker*2 years), p-value | 0.94 | 0.357 | 0.221 | 0.489 | 0.267 | 0.654 |
|  | Interaction term (marker*3 years), p-value |  |  |  | 0.24 |  |  |
|  | All | 0.03 (-0.48, 0.54) | 0.14 (-0.31, 0.58) | -0.33 (-1.14, 0.47) | 0 (-0.36, 0.36) | 0.46 (-0.01, 0.92) | 0.02 (-0.46, 0.5) |
|  | 1 year | 0.06 (-0.5, 0.62) | 0.01 (-0.51, 0.53) | -0.86 (-1.57, -0.14) | 0.14 (-0.38, 0.66) | 0.03 (-0.44, 0.5) | -0.14 (-0.6, 0.32) |
|  | 2 years | 0.24 (-0.6, 1.09) | 0.49 (-0.33, 1.31) | 0.28 (-1.2, 1.77) | -0.12 (-0.64, 0.4) | 0.91 (0.12, 1.69) | 0.32 (-0.69, 1.33) |
|  | 3 years |  |  |  | -0.37 (-1.14, 0.4) |  |  |
| fCLP | Interaction term (marker*2 years), p-value | 0.835 | 0.75 | 0.975 | 0.221 | 0.408 | 0.709 |
|  | Interaction term (marker*3 years), p-value |  |  |  | 0.568 |  |  |
|  | All | -0.47 (-2.25, 1.3) | -0.04 (-1.58, 1.5) | 1.07 (-1.79, 3.94) | 0.27 (-1.09, 1.64) | 0.1 (-1.58, 1.79) | 0.2 (-1.52, 1.93) |
|  | 1 year | -0.83 (-2.83, 1.17) | 0.19 (-1.67, 2.06) | 1.09 (-1.52, 3.69) | -0.34 (-2.2, 1.52) | 1.16 (-0.55, 2.86) | 0.23 (-1.45, 1.92) |
|  | 2 years | -1.15 (-4.05, 1.75) | -0.95 (-3.75, 1.84) | 1.22 (-3.87, 6.3) | 1.22 (-0.48, 2.91) | -0.63 (-3.46, 2.19) | -0.03 (-3.38, 3.32) |
|  | 3 years |  |  |  | 0.43 (-3.81, 4.68) |  |  |
| aPGHS | Interaction term (marker*2 years), p-value | 0.393 | 0.883 | 0.977 | 0.179 | 0.5 | 0.45 |
|  | Interaction term (marker*3 years), p-value |  |  |  | 0.08 |  |  |
|  | All | -0.11 (-0.72, 0.5) | 0.03 (-0.5, 0.56) | -0.84 (-1.8, 0.12) | 0.03 (-0.4, 0.46) | 0.38 (-0.18, 0.94) | -0.09 (-0.66, 0.48) |
|  | 1 year | 0.13 (-0.52, 0.78) | 0 (-0.6, 0.61) | -0.91 (-1.75, -0.08) | 0.33 (-0.27, 0.94) | 0.12 (-0.43, 0.66) | -0.04 (-0.58, 0.49) |
|  | 2 years | -0.21 (-1.28, 0.87) | 0.19 (-0.83, 1.22) | -0.87 (-2.75, 1.02) | -0.27 (-0.94, 0.39) | 0.78 (-0.24, 1.8) | -0.19 (-1.44, 1.05) |
|  | 3 years |  |  |  | -0.44 (-1.32, 0.45) |  |  |
| aCLP | Interaction term (marker*2 years), p-value | 0.218 | 0.117 | 0.066 | 0.898 | 0.141 | 0.147 |
|  | Interaction term (marker*3 years), p-value |  |  |  | 0.766 |  |  |
|  | All | -0.47 (-1.39, 0.44) | -0.39 (-1.18, 0.4) | -0.56 (-2.04, 0.93) | 0.16 (-0.55, 0.87) | -0.63 (-1.48, 0.23) | -0.23 (-1.11, 0.65) |
|  | 1 year | -0.09 (-1.11, 0.93) | 0.02 (-0.93, 0.97) | 0.88 (-0.44, 2.2) | 0.26 (-0.69, 1.21) | 0.47 (-0.38, 1.31) | 0.41 (-0.42, 1.24) |
|  | 2 years | -1.48 (-2.97, 0) | -1.41 (-2.85, 0.03) | -2.23 (-4.83, 0.36) | 0.19 (-0.69, 1.07) | -1.49 (-2.88, -0.09) | -1.29 (-2.97, 0.39) |
|  | 3 years |  |  |  | 0.59 (-1.81, 3) |  |  |
